# EPR Spectroscopy: A Powerful Tool to Analyze Supramolecular Host•Guest Complexes of Stable Radicals with Cucurbiturils

**DOI:** 10.3390/molecules25040776

**Published:** 2020-02-11

**Authors:** Fengbo Liu, Hakim Karoui, Antal Rockenbauer, Simin Liu, Olivier Ouari, David Bardelang

**Affiliations:** 1 The State Key Laboratory of Refractories and Metallurgy, School of Chemistry and Chemical Engineering, Wuhan University of Science and Technology, Wuhan 430081, China; lfb1993@126.com; 2 Aix Marseille Univ, CNRS, ICR, 13013 Marseille, France; hakim.karoui@univ-amu.fr; 3 Institute of Materials and Environmental Chemistry, Research Centre for Natural Sciences, 1117 Budapest, Hungary; rockenbauer.antal@ttk.mta.hu; 4 Department of Physics, Budapest University of Technology and Economics, Budafoki ut 8, 1111 Budapest, Hungary

**Keywords:** EPR spectroscopy, supramolecular chemistry, host•guest chemistry, nitroxides, cucurbiturils

## Abstract

Stable organic free radicals are increasingly studied compounds due to the multiple and unusual properties imparted by the single electron(s). However, being paramagnetic, classical methods such as NMR spectroscopy can hardly be used due to relaxation and line broadening effects. EPR spectroscopy is thus better suited to get information about the immediate surroundings of the single electrons. EPR has enabled obtaining useful data in the context of host•guest chemistry, and a classical example is reported here for the stable (2,2,6,6-tetramethyl-4-oxo-piperidin-1-yl)oxyl or 4-oxo-TEMPO nitroxide (**TEMPONE**) inside the macrocycle host cucurbit[7]uril (CB[7]). Generally and also observed here, a contraction of the spectrum is observed as a result of the reduced nitrogen coupling constant due to inclusion complexation in the hydrophobic cavity of the host. Simulations of EPR spectra allowed determining the corresponding binding constant pointing to a weaker affinity for CB[7], compared to TEMPO with CB[7]. We complement this work by the results of EPR spectroscopy of a biradical: bis-TEMPO-bis-ketal (**bTbk**) with cucurbit[8]uril (CB[8]). Initial investigations pointed to very weak effects on the spectrum of the guest and incorrectly led us to conclude an absence of binding. However, simulations of EPR spectra combined with NMR data of reduced bTbk allowed showing inclusion complexation. EPR titrations were performed, and the corresponding binding constant was determined. ^1^H NMR spectra with reduced bTbk suggested a shuttle mechanism, at nearly one equivalent of CB[8], for which the host moves rapidly between two stations.

## 1. Introduction

**Scheme 1:**
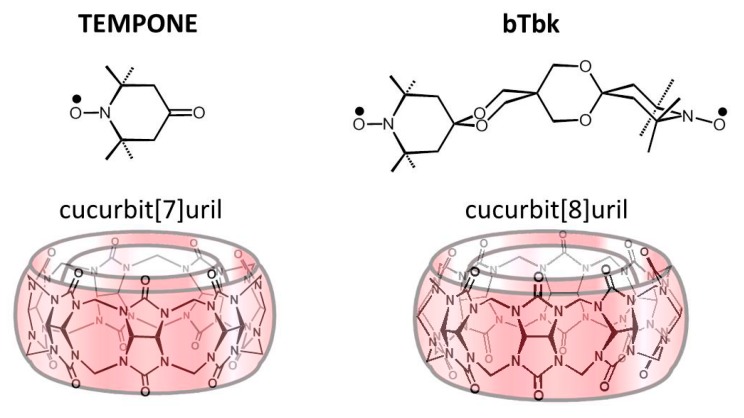
Structures of nitroxides **TEMPONE** and bis-TEMPO-bis-ketal (**bTbk**), and of cucurbiturils CB[7] and CB[8].

Since the discovery of the first organic stable radical by Gomberg in 1900 [1], radical chemistry has dramatically expanded with initial intense focus on mastering the structures [2] and the life times [3] of transient species before using transient or stable free radicals in applications ranging from living polymerization [4], to batteries [5], or more recently, for dynamic nuclear polarization (DNP) [6,7]. Free radicals are intimately related to EPR spectroscopy since this technique is specially focused on investigating the single electron(s) carried by several types of compounds. EPR spectra contain a wealth of information, which, correctly extracted, can give users access to crucial information such as stability, structure, or dynamics, in various liquid or solid environments [8]. Supramolecular chemistry principally features diamagnetic compounds, but paramagnetic ones are expected to enable access to new types of applications including molecular magnetic switches [9], dynamic covalent systems [10], or tracers for imaging by stabilizing free radicals in reducing conditions. In the context of host•guest chemistry, the guests are almost exclusively [11] the radicals, and they are included in macrocyclic hosts. Several reviews [12,13,14] have already documented host•guest complexes featuring free radicals and several families of hosts including calixarenes, cyclodextrins, and cucurbiturils. This latter family of pumpkin-shape macrocycles [15,16,17,18,19] possess unique properties, in a broader context, such as ultrahigh binding [20,21], gas adsorption [22], or drug encapsulation and release [23,24]. With cucurbit[*n*]urils (CB[*n*]), the main radicals studied are nitroxides [25], in the majority of cases stable ones. In a seminal work, Lucarini and coworkers studied the cucurbit[7]uril (CB[7]) cavity by EPR spectroscopy using several nitroxides [26]. Later, Kaifer and coworkers showed how CB[*n*] could modulate the extent of spin exchange between covalently linked nitroxides [27]. We also discovered in 2009 [28], thanks to EPR spectroscopy, the formation of triangular 3:3 host:guest assemblies of 1:1 CB[8]:nitroxide complexes, at the same time as Lucarini and coworkers [29] and the teams of Ottaviani, Ramamurthy, and Turro [30]. EPR spectroscopy was key in this finding since it gave a clear spectral signature of the triangular assembly that could hardly be found by other techniques. We recently showed that, provided the guest possesses the right structure, diamagnetic triangles of CB[8] could also be produced and hence studied by NMR [31]. Since CB[*n*] are almost exclusively soluble in water, nitroxide guest recognition is monitored using EPR by comparing the spectrum of the radical alone to that in the presence of the host in aqueous solutions. Often, a contraction of the spectrum is observed as a result of the guest experiencing a more hydrophobic environment upon inclusion in CB[*n*] (reduced nitrogen coupling constant *a*_N_), and the high-field line is broadened due to reduced tumbling (i.e., reduced mobility because of a larger molecular weight) [12,13,14,26]. This way, inclusion of the nitroxide function carrying the single-electron in the host cavity is believed to be important to obtain significant changes in EPR spectra. Here, we illustrate this concept using the 4-oxo-TEMPO (or **TEMPONE**, Scheme 1) nitroxide with CB[7].

This host•guest system has been briefly investigated before [32] in the context of improved resistance to bioreduction provided by CB[*n*] and unusual rotational dynamics, but with a simple mention for the order of magnitude of the binding constant (~10^3^ M^−1^). We describe here the EPR titration and simulations of EPR spectra allowing determining the binding constant. On the other hand, dinitroxide biradicals exhibiting spin exchange or not have started to be widely investigated for applications in DNP [6,7], and cyclodextrins proved to be interesting macrocycles to modulate polarization transfer [33]. In this context, we wanted to see if inclusion complexation could occur between a cucurbituril and a representative dinitroxide used for DNP: the stable dinitroxide of TEMPO, **bTbk** [34], to enhance its water solubility for perspective work in DNP.

## 2. Results

The first case investigated is the one of the nitroxide **TEMPONE** for which the corresponding EPR spectrum is known to exhibit three sharp lines.

### 2.1. Complexation between TEMPONE and CB[7]

**Figure 1:**
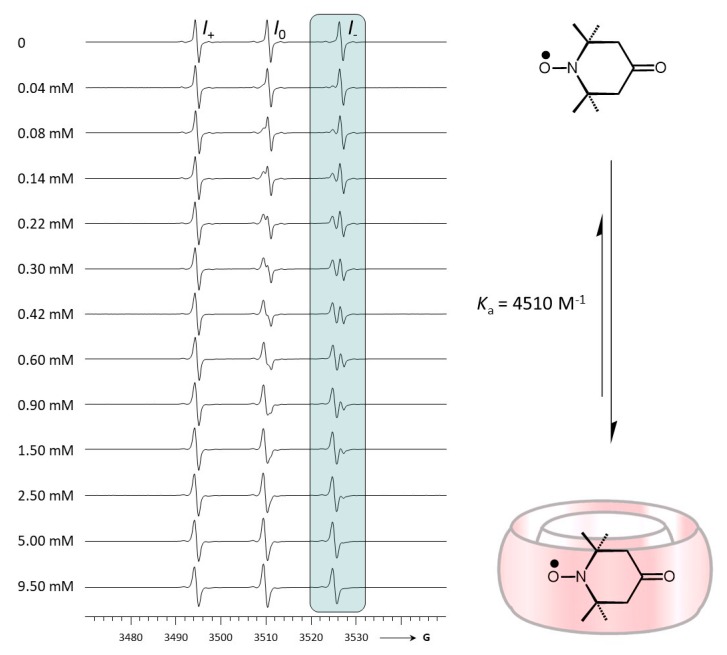
EPR titration of **TEMPONE** (0.1 mM) with CB[7] (concentration on the left) in water and proposed inclusion complexation (right).

The EPR spectrum of **TEMPONE** in water is characterized by three main lines with a width at half-height (Wahh) of ~0.8 G (Figure 1). Two small ^13^C satellite lines can be seen just next to each of the three main lines as a result of a weak coupling with isotopically rare ^13^C (nuclear spin = 1/2).

Twenty-five spectra were recorded with increased concentrations of CB[7] illustrating the gradual inclusion of **TEMPONE** in CB[7] (Figure 1 and Figures S1 to S25). The presence of additional lines especially at high field for intermediate host:guest ratios indicated slow exchange with respect to the EPR timescale. Above 5 mM of CB[7], no further change could be observed, and the spectrum was that of the included radical featuring reduced *a*_N_ (from ~16.0 G for **TEMPONE** alone to ~15.3 G in the complex) typical of a switch to a more hydrophobic surrounding. These results were in line with previous reports of TEMPO in CB[7] suggesting perpendicular position of the nitroxide group with respect to the *C*_7_ axis of CB[7] [26,28]. All the spectra were then simulated thanks to a 2D EPR program [35] (Figures S1 to S28) enabling testing several hypotheses of binding. The simplest case assuming a host:guest 1:1 binding afforded a binding constant *K*_a_ = 4510 M^−1^ (Table 1) with a regression coefficient of 0.9918.

Controls using reduced **TEMPONE** with CB[7] by ^1^H NMR afforded a similar binding constant of *K*_a_ = 3960 M^−1^ (slightly weaker due to the more polar feature expected for the hydroxylamine of reduced **TEMPONE**, Figure S29). However, simulated EPR spectra at high CB[7] concentrations showed better agreement with experimental ones when a 2:1 complex was considered (with two CB[7]). Previous reports mentioned the possibility for cucurbiturils to aggregate in water [28,29,30,31,36,37,38,39,40], but there is only a slight improvement in the agreement between simulated spectra and experimental ones (regression coefficient of 0.9955) assuming this model; other techniques would be necessary to confirm this possibility. As mentioned above, a decrease in *a*_N_ was associated with a more hydrophobic surrounding for the nitroxide group, while the observed decrease in *a*_C_ also indicated a redistribution of spin densities on the corresponding ring, together with a probable freeze in dihedral angles due to the inclusion.

### 2.2. Complexation between bTbk and CB[8]

**Figure 2:**
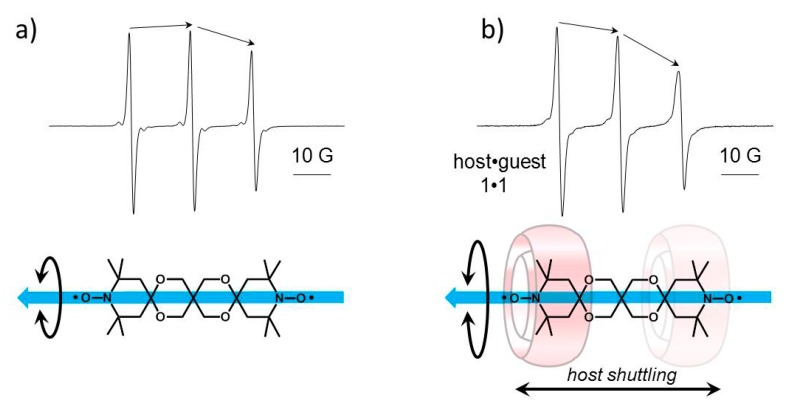
EPR spectra of **bTbk** (50 μM) without (**a**) and with CB[8] (1.1 mM, (**b**) and the proposed host position and shuttling based on EPR and NMR (see the text).

In **bTbk**, the two TEMPO residues carrying the single electrons are too far for spin exchange, and they thus behave magnetically independently, giving rise to EPR spectra with three lines as for TEMPO alone (Figure 2a). **bTbk** is still an excellent polarizing agent for DNP-enhanced applications, but is hardly soluble in water [34]. Cyclodextrins have previously been used to improve the solubility of **bTbk** for DNP applications [33], and we reasoned that CB[7] or CB[8] could be another interesting option for this purpose.

However, **bTbk** is a larger molecule compared to **TEMPONE**, and a different binding mode is expected for its association with CB[7] or CB[8]. While CB[7] showed almost undetectable effects, the addition of CB[8] slightly impacted the EPR spectrum of **bTbk** in water. The *a*_N_ coupling constant was slightly reduced (by an ~0.17 G average value, Table 2), in stark contrast with the usual behaviors (i.e., Δ*a*_N_ = 0.7 G for **TEMPONE** with CB[7]).

This small reduction of *a*_N_ upon CB[*n*] addition initially led us to conclude an absence of binding or a weak external binding. However, the slight broadening of the high-field line was significant as the *I*_-_/*I*_0_ ratio (Table 2), but we could not explain it until we realized that the inclusion of **bTbk** in CB[8] would leave the EPR responsive N-O• bonds significantly bulk exposed, because the TEMPO fragments cannot be included as for **TEMPONE** in the **TEMPONE**•CB[7] complex. Because **bTbk** is too large to be fully included in a CB, the guest has to orientate such that the TEMPO fragments of **bTbk** bind in the CB cavity with the nitroxide group parallel to the *C*_8_ symmetry axis of the host. In this geometry (Figure 2b), the N-O• bonds are not facing the cavity walls anymore and become able to interact with water molecules, even if the guest is included in the host. In this particular case, the best evidence comes from the broader high-field line of the EPR spectrum (Figure 2b), suggesting a slower molecular tumbling caused by a larger molecular weight.

By analyzing a series of 36 EPR spectra of **bTbk** with increasing host concentrations (Figures S30 to S68) by the same 2D simulation program as that used for **TEMPONE [35]**, we could determine the respective concentrations of each species at relevant host concentrations and evaluate the corresponding binding constant (*K*_a_ 1:1 model in Table 2). In this first model, averaged effects were assumed (two TEMPO groups with the same parameters). However, ^1^H NMR spectra of reduced **bTbk** suggested a shuttling process for CB[8] (Figure S69). Indeed, ^1^H NMR spectroscopy of **bTbk** (0.5 mM) with excess ascorbic acid (used to reduce the N-O• bonds into diamagnetic N-OH bonds) showed that, with 1.25 equiv. of CB[8], the signals of all methyl groups were shifted upfield by 0.29 ppm (Figure S69). This was in line with the slightly reduced *a*_N_ value of **bTbk** observed with CB[8] (<Δ*a*_N_> = 0.17 G). For binding the two reduced TEMPO fragments, one would expect a necessary quantity of two CB[8]. Because only 1.25 equiv. of CB[8] was enough to shield the signals of all methyl groups, we proposed a 1:1 binding mode in which CB[8] would shuttle between two TEMPO stations (Figure 2b). ^1^H NMR spectra at 2 equiv. of CB[8] were identical to those at 1.25 equiv. of CB[8] (Figure S69). Hence, even if with 1 equiv. of CB[8], a shuttle movement of CB[8] was suspected; with 2 equiv. of the host, a 1:2 complex was possible (Figure S69). However, in the conditions used to record EPR spectra ([**bTbk**] = 0.05 mM), 1:1 complexes mainly formed.

We thus had to consider another model for simulations of EPR spectra. In a second model, the first TEMPO group had EPR parameters fixed and corresponding to non-included TEMPO, and the second TEMPO group had its parameters fitted (complexation by CB[8], overall regression parameter 0.9959). With the second “shuttle” model (Table 2), the obtained binding constant of 20 900 M^−1^ was consistent with the value obtained by NMR. Indeed, the NMR spectrum at ~1.1 equiv. of CB[8] (Figure S69) showed signals for methyl groups assigned to free guest and the 1:1 complex in a ratio of ~ 5/19 respectively (79% of complexed guest). Assuming that the binding constant of reduced **bTbk** was similar to that of **bTbk** (*K*_a_ ≈ 20 900 M^−1^), this would give 77% respectively of 1:1 complex in the conditions of NMR, while the value determined from ^1^H NMR was 79%. The proposed binding constant of **bTbk** toward CB[8] determined by EPR was consistent with ^1^H NMR spectra of reduced **bTbk**. Controls by EPR of **bTbk** with CB[8] using a competitor (amantadine hydrochloride) that was known to have a very high affinity for CB[8] showed free **bTbk** in solution (Figure S70), supporting an associative mechanism of **bTbk** with CB[8] in the absence of competitor. Finally, simulations provided relaxation parameters α, β, and γ (see Supporting Information), which were related to the tumbling of the molecule. They were determined from the time dependent perturbation theory [41,42] from the anisotropy of *g* and a_N_ hyperfine tensors. α depends on the anisotropy of *g*, β on the product of *g* and a_N_ tensors, while γ is determined from the anisotropy of the a_N_ tensor [41,42]. The rotational tumbling can average the tensors, but the scatter of resonance position can produce line broadening expressed by the relaxation parameters. If the rotational tumbling becomes asymmetric, averaging of tensors will produce different values, which strongly influences the broadening effect. In this case, compared to the free guest (or free TEMPO group), relaxation parameters for the uncomplexed TEMPO group did not change much except for α, which was about doubled. However, significant differences were found for the complexed TEMPO group. First, a decrease in *a*_N_ of 0.34 G was observed, typical of a nitroxide inclusion in CB[8] with the N-O• bond relatively bulk exposed [31]. Then, while the α value increased by ~50%, β and γ were multiplied by a factor of ~5, suggesting a tumbling on this side, largely different from the uncomplexed side. The presence of CB[8] surrounding the complexed TEMPO group expectedly hampered movements on this side of **bTbk**, while the tumbling remained about similar on the other side (totally bulk-exposed TEMPO group).

## 3. Discussion

The association of **TEMPONE** with CB[7] is typical of the “general case” we have frequently observed for which the nitrogen coupling constant is reduced as a result of the nitroxide bond experiencing a more hydrophobic surrounding. We observed this behavior for a variety of nitroxides in the presence of several types of cyclodextrins [43,44,45] and of cucurbiturils [28,31,39]. Besides kinetic [46] and thermodynamic [47] parameters, which can sometimes be extracted from EPR experiments, binding constants are important parameters to assess the affinity of a guest for a host. The binding constant of **TEMPONE** for CB[7] (*K*_a_ = 4510 M^−1^) was lower than that of TEMPO for CB[7], which was ~10^4^ M^−1^ [26,28]. As both guests bonded in CB[7] with the nitroxide groups perpendicular to the *C*_7_ axis of the host, the lower value determined for **TEMPONE** could be ascribed to the ketone function, which may be more difficult to desolvate to enter the cavity of CB[7] compared to the corresponding -CH_2_- group of TEMPO.

Contrary to **TEMPONE**, **bTbk** is a much larger molecule that cannot be entirely complexed by CB[7] or CB[8]. When one TEMPO fragment enters a sufficiently large CB cavity (at least that of CB[8]), the remaining bis-ketal-TEMPO part remains bulk-exposed, but not only (Figure 2b). The N-O• bond of the included TEMPO fragment is also exposed to water because, due to the bulkiness of the bis-ketal-TEMPO remaining group, the included N-O• bond cannot be placed like that of **TEMPONE** in CB[7] (i.e., facing the host cavity walls). This was the reason for the only slight effects observed in EPR spectra. The largest changes in these cases were expected to be due to reduced *a*_N_ coupling constants, but these were very sensitive to solvent polarity. If the two N-O• bonds remained bulk-exposed, then no large changes could be expected besides an increase in molecular weight and lower tumbling. This indicated that, slight effects on EPR spectra of paramagnetic guests after the addition of relevant hosts did not necessarily mean a lack of binding, and researchers should be cautious when analyzing EPR spectra. The solubility of **bTbk** in water was around 50 μM, and CB[8] did not increase it very largely. However, these investigations allowed finding that molecular shuttles using cucurbiturils were not limited to CB[6] [48] or CB[7] [49,50,51] and that CB[8] could also behave as a functional ring in water-soluble molecular shuttles provided the guest had the right structure.

## 4. Materials and Methods 

The methods and protocols used in this work are mentioned in this paper and referred to in the References section. More details can be found in the Supporting Information.

## 5. Conclusions

The first case investigated (binding of **TEMPONE** in CB[7]) was efficiently monitored by EPR spectroscopy and allowed to determine a previously undescribed binding constant of *K*_a_ = 4510 M^−1^. Additionally, EPR allowed identifying a new supramolecular complex between **bTbk** and CB[8], initially thought to be weak or not forming, due to the very slight changes observed on EPR spectra upon host addition. EPR titrations and simulations of EPR spectra allowed proposing a binding constant *K*_a_ = 20 900 M^−1^. ^1^H NMR of the reduced radical (reduced **bTbk**) suggested a shuttling mechanism, which was accounted for in simulations of EPR spectra. Besides the modulation of the properties imparted by the single electron of the N-O• bonds, we believe that this kind of complex could have relevance for investigating the dynamics of molecular machines [52,53] such as supramolecular rotors and shuttles.

## Figures and Tables

**Table 1:** EPR parameters for **TEMPONE** and the host:guest 1:1 complex. Wahh, width at half-height.

	TEMPONE	TEMPONE•CB[7]
*g*	2.0055 ^a^	2.0060 ^a^
*a*_N_/G	15.99 ± 0.05 ^b^	15.29 ± 0.05 ^b^
*a*_C_/G	6.0 ± 0.5 ^b^	4.7 ± 0.5 ^b^
Wahh/G	0.82 ± 0.05 ^a^	0.96 ± 0.06 ^a^
*I*_-_/*I*_0_	0.92 ^a^	0.85 ^a^
*K*_a_/M^−1^	-	4510 ± 500 ^b^

^a^ From experimental spectra. ^b^ From simulations.

**Table 2:** EPR parameters for **bTbk** and for the **bTbk**•CB[8] complex. Binding constants based on simulations of EPR spectra (see the text).

	bTbk	bTbk•CB[8]
*g*	2.0055 ^a^	2.0056 ^a^
*a*_N_/G	16.59 ± 0.05 ^b^	16.25 ± 0.05 and 16.59 ± 0.05 ^b^
*a*_C_/G	5.8 ±0.5 ^b^	6.0 ± 0.5 and 5.8 ± 0.5 ^b^
Wahh/G	1.18 ± 0.05 ^a^	1.44 ± 0.10 ^a^
*I*_-_/*I*_0_	0.78 ^a^	0.67 ^a^
*K*_a 1:1 model_/M^−1^	-	36 600 ±5000 M^−1, b^
*K*_a shuttling model_/M^−1^	-	20 900 ± 5000 M^−1, b^

^a^ From experimental spectra. ^b^ From simulations (see Supporting Information).

## Supplementary Materials

Simulations of EPR spectra and ^1^H NMR spectra are available online.
