# Supplementary material for: EPR Spectroscopy: A Powerful Tool to Analyze Supramolecular Host•Guest Complexes of Stable Radicals with Cucurbiturils
Source: Molecules. 2020 Feb 11;25(4):776. doi: 10.3390/molecules25040776 (PMC7070855; doi:10.3390/molecules25040776)
Supplement: Supplementary file 1 [file molecules-25-00776-s001.pdf]

Supporting Information

EPR spectroscopy: a powerful tool to analyze supramolecular Host•Guest complexes of stable radicals with Cucurbiturils.

Fengbo Liu, Hakim Karoui, Antal Rockenbauer, Simin Liu, Olivier Ouari and David Bardelang

Table of Contents

1/ Chemical compounds --- S2

2/ NMR Measurements --- S2

3/ EPR Measurements --- S2

4/ EPR titration and simulations for **TEMPONE** with CB[7] ------------------------------------ S3

5/ NMR spectra of reduced **TEMPONE** with CB[7] --- S13

6/ EPR titration and simulations for **bTbk** with CB[8] -- S14

7/ NMR spectra of reduced **bTbk** with CB[8] --- S28

8/ Competition experiments (EPR) for the binding of **bTbk** with CB[8] ---------------------- S29

9/ References -- S29

Experimental procedures

1/ Chemical compounds. The **TEMPONE** radical was from Sigma (90-95%) and used as received. **bTbk** was prepared as previously reported[1]. CB[7] was prepared following usual procedures[2],[3] refluxing glycoluril and solid aldehyde in HCl at 125°C before separation using water and MeOH/water mixtures according to the solubility of CB[7]. Then, CB[7] was purified by chromatography on silica gel eluting with a mixture of water / acetic acid / formic acid = 10 / 10 / 1. Neutral CB[7] (pH ~7) was obtained by repeatedly evaporating water from aqueous solutions of the column-purified CB[7] under reduced pressure until the pH was 7. CB[8] was prepared as previously reported[4]. Ascorbic acid and amantadine hydrochlorides were from Sigma-Aldrich (highest purity available) and used as received.

2/ NMR Measurements**.** NMR spectra were acquired on a BRUKER Avance III nanobay – 300 spectrometer (^{1}H-NMR 300.13 MHz). D_2O was used as the solvent and a watergate sequence (water suppress) was used when relevant (altering signals and integrals near 4.7 ppm). Ascorbic acid was added to D_2O solutions to reduce the nitroxide and get corresponding hydroxylamines which are diamagnetic such as making the NMR analysis relevant.

3/ EPR Measurements**.** EPR measurements were performed on a Bruker Elexsys E500 spectrometer operating at 9.4 GHz (X-band) using the following parameters: microwave power 10 mW, receiver gain 75 dB, time constant 0.128 s, sweep width 80 G, sweep time 30s, modulation amplitude of 1 G and 1 scan.

For the titration of **TEMPONE** with CB[7], two stock solutions of host were prepared in distilled water at 10 mM and at 1 mM as well as a stock solution of guest at 2 mM in distilled water. Final volumes of 100 μL were targeted, adding relevant volumes of appropriate stock solutions to reach the target concentrations fixing that of the guest at 0.1 mM final concentration (5 μL of stock solution) and completing with distilled water when necessary. After mixing, 50 μL were taken in capillary tubes before introduction in the sample holder and record of EPR spectra.

For the titration of **bTbk** with CB[8], a stock solution of **bTbk** was prepared at 0.1 mM in distilled water repeating 3 cycles of heating/ultrasound/vortex to ensure sample dissolution. 50 μL were taken up and mixed with relevant volumes of stock solutions of CB[8] and of distilled water to reach 100 μL of final volume and so 50 μM final concentration of **bTbk** (0.1 mM final concentration in radical). Several stock solutions of CB[8] at different concentrations were assessed with heating/ultrasound exposure prior to picking up the right volumes and addition in eppendorfs containing appropriate volumes of the **bTbk** solution and of water. 100 μL final volumes were targeted and 50 μL of solutions were taken up in capillary tubes before introduction in the sample holder and record of EPR spectra.

4/ EPR titration (black spectra) and simulations (red spectra) for TEMPONE with CB[7].

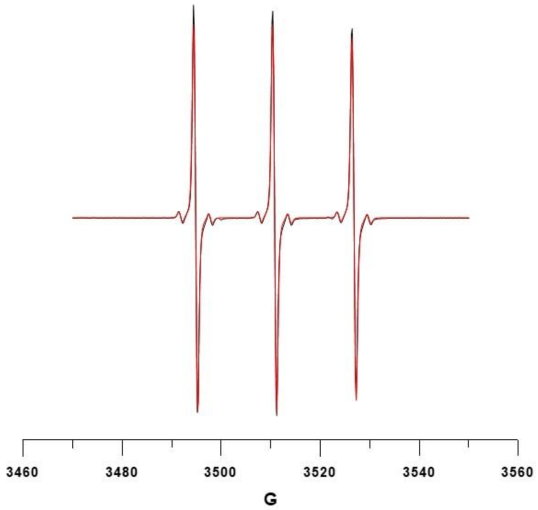

Figure S1. Experimental and simulated EPR spectra of **TEMPONE** (0.1 mM) with CB[7] (0 mM) in water.

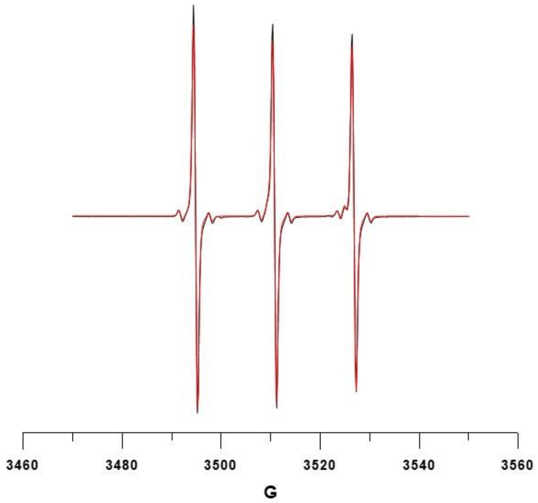

Figure S2. Experimental and simulated EPR spectra of **TEMPONE** (0.1 mM) with CB[7] (0.02 mM) in water.

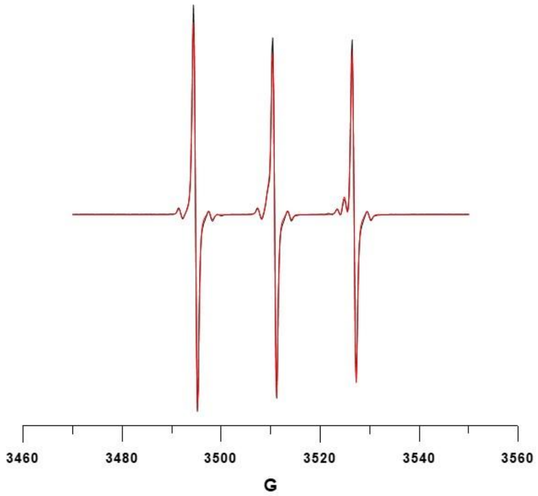

Figure S3. Experimental and simulated EPR spectra of **TEMPONE** (0.1 mM) with CB[7] (0.04 mM) in water.

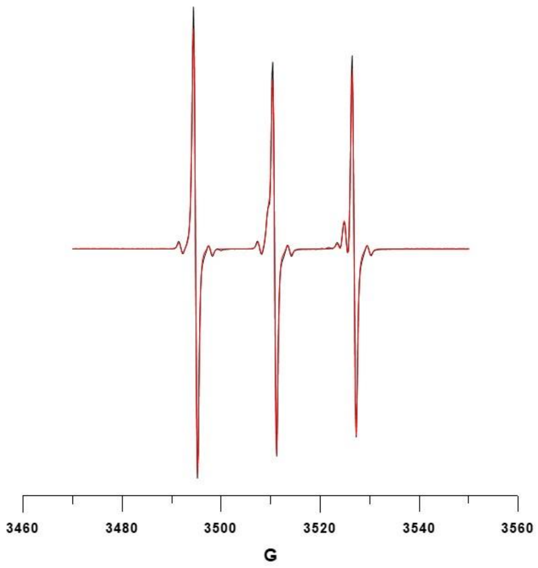

Figure S4. Experimental and simulated EPR spectra of **TEMPONE** (0.1 mM) with CB[7] (0.06 mM) in water.

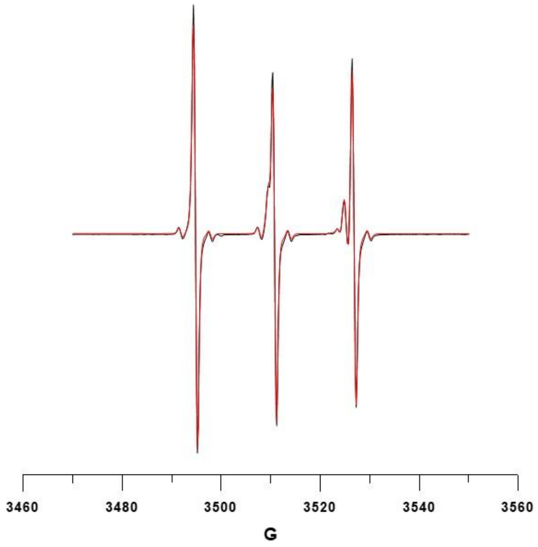

Figure S5. Experimental and simulated EPR spectra of **TEMPONE** (0.1 mM) with CB[7] (0.08 mM) in water.

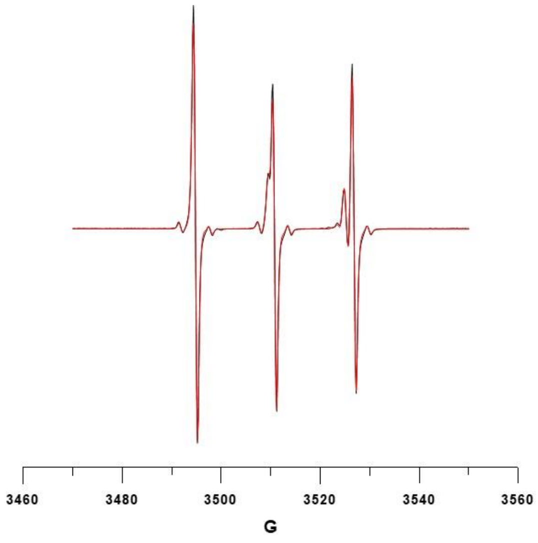

Figure S6. Experimental and simulated EPR spectra of **TEMPONE** (0.1 mM) with CB[7] (0.10 mM) in water.

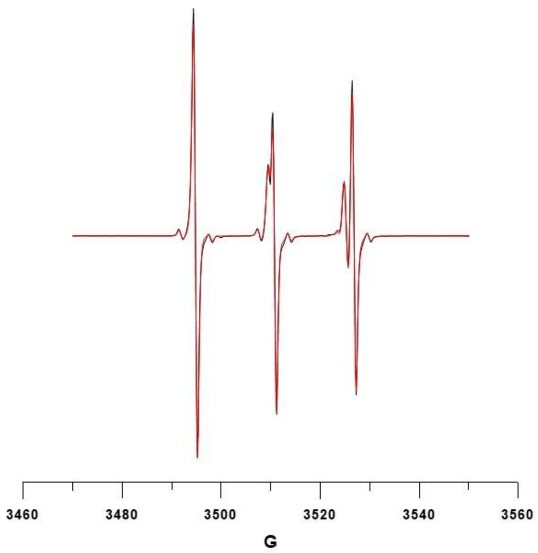

Figure S7. Experimental and simulated EPR spectra of **TEMPONE** (0.1 mM) with CB[7] (0.14 mM) in water.

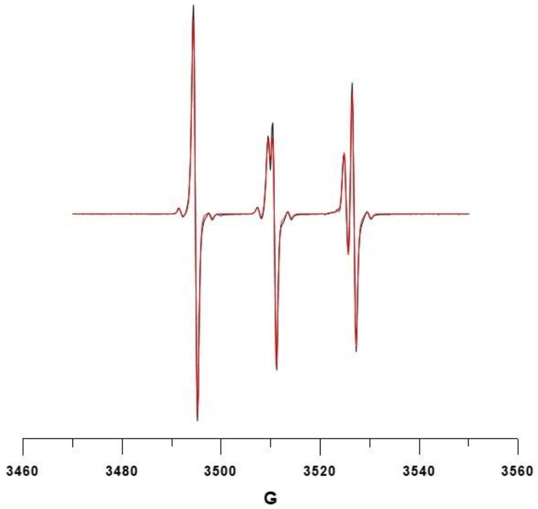

Figure S8. Experimental and simulated EPR spectra of **TEMPONE** (0.1 mM) with CB[7] (0.18 mM) in water.

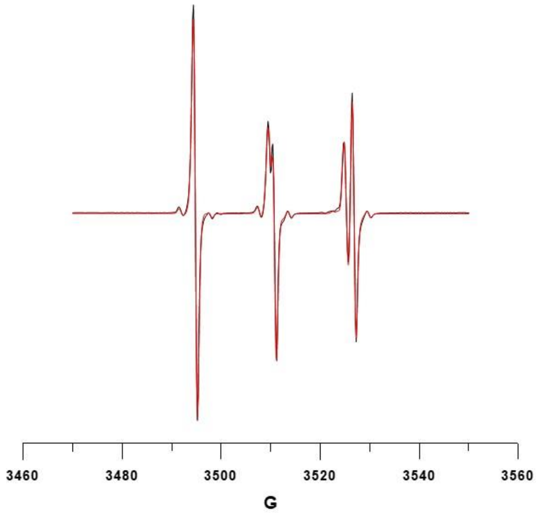

Figure S9. Experimental and simulated EPR spectra of **TEMPONE** (0.1 mM) with CB[7] (0.22 mM) in water.

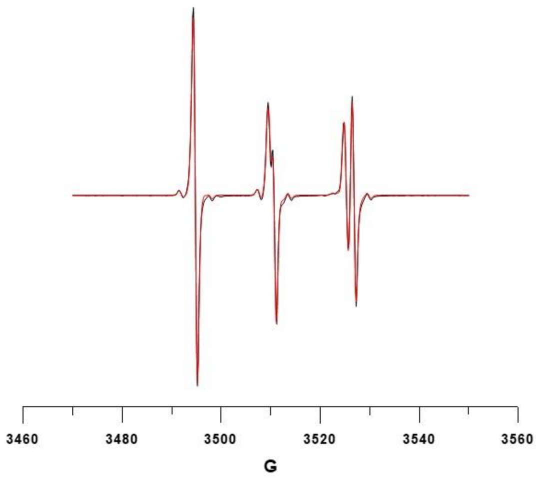

Figure S10. Experimental and simulated EPR spectra of **TEMPONE** (0.1 mM) with CB[7] (0.26 mM) in water.

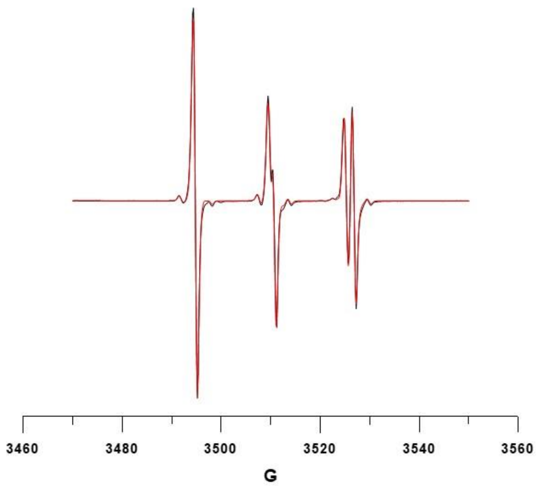

Figure S11. Experimental and simulated EPR spectra of **TEMPONE** (0.1 mM) with CB[7] (0.30 mM) in water.

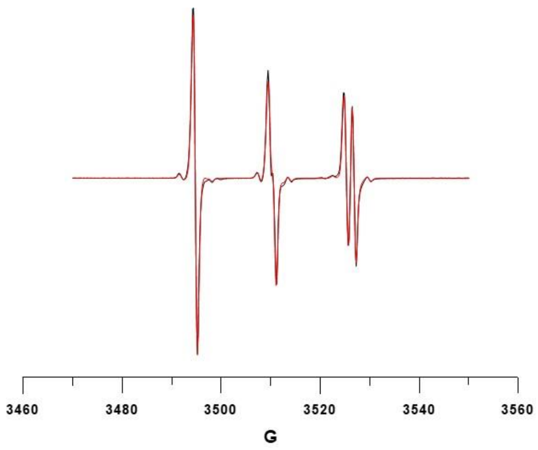

Figure S12. Experimental and simulated EPR spectra of **TEMPONE** (0.1 mM) with CB[7] (0.36 mM) in water.

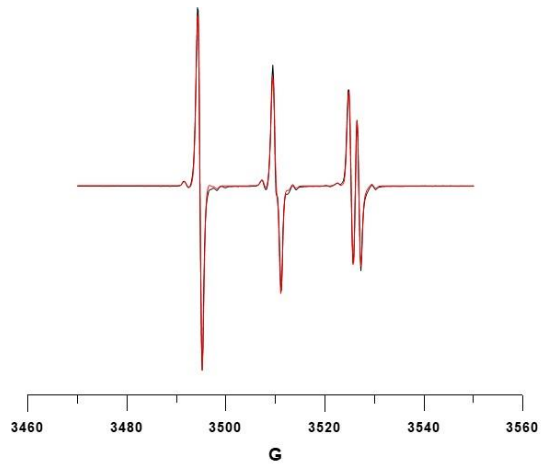

Figure S13. Experimental and simulated EPR spectra of **TEMPONE** (0.1 mM) with CB[7] (0.42 mM) in water.

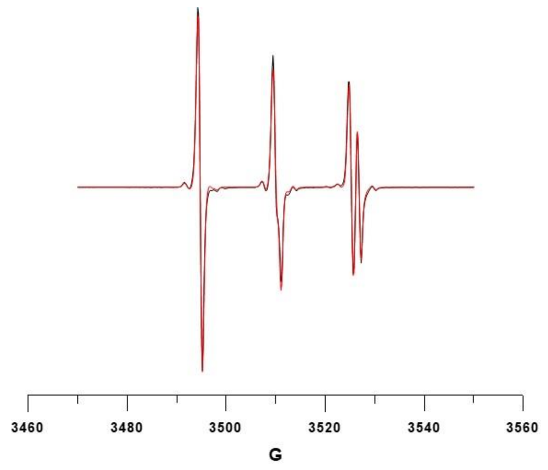

Figure S14. Experimental and simulated EPR spectra of **TEMPONE** (0.1 mM) with CB[7] (0.50 mM) in water.

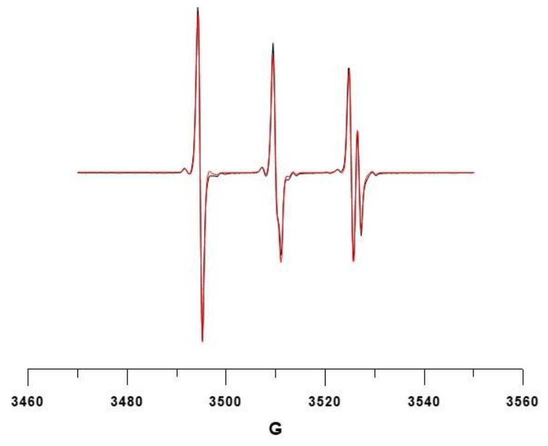

Figure S15. Experimental and simulated EPR spectra of **TEMPONE** (0.1 mM) with CB[7] (0.60 mM) in water.

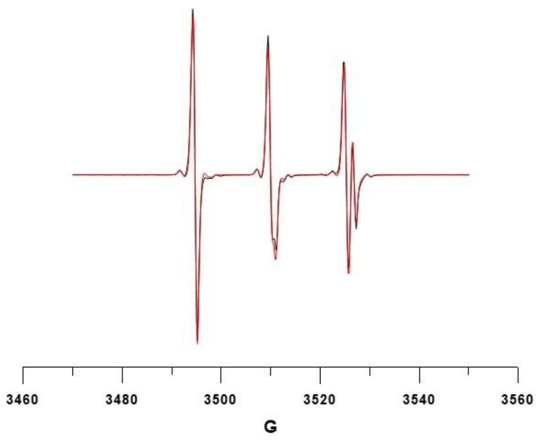

Figure S16. Experimental and simulated EPR spectra of **TEMPONE** (0.1 mM) with CB[7] (0.75 mM) in water.

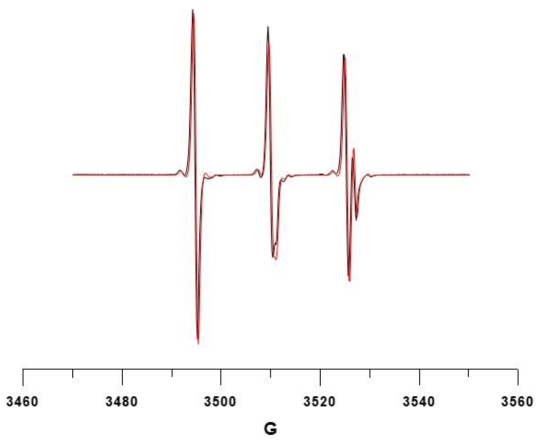

Figure S17. Experimental and simulated EPR spectra of **TEMPONE** (0.1 mM) with CB[7] (0.90 mM) in water.

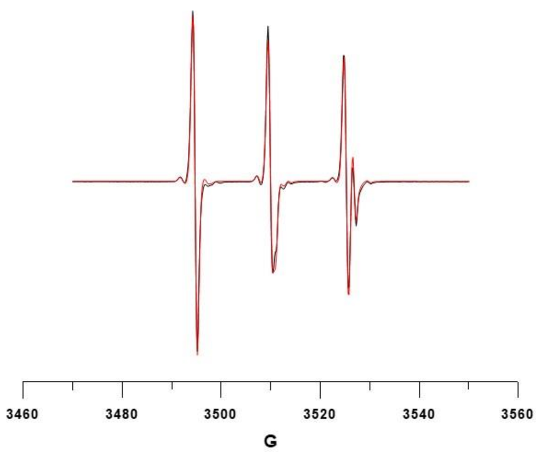

Figure S18. Experimental and simulated EPR spectra of **TEMPONE** (0.1 mM) with CB[7] (1.00 mM) in water.

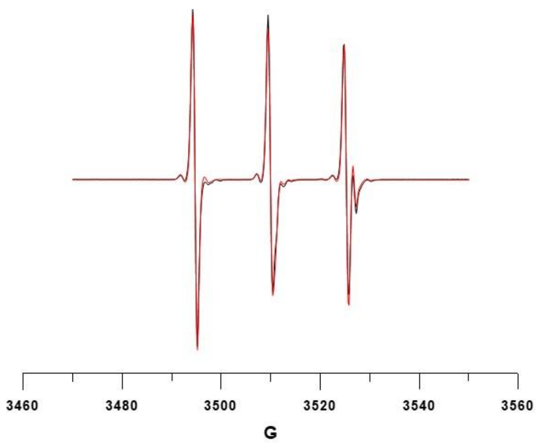

Figure S19. Experimental and simulated EPR spectra of **TEMPONE** (0.1 mM) with CB[7] (1.50 mM) in water.

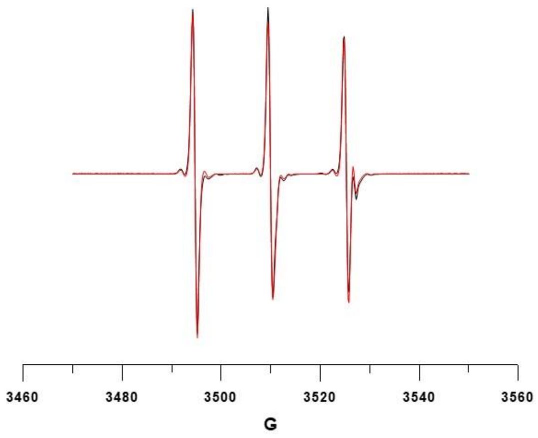

Figure S20. Experimental and simulated EPR spectra of **TEMPONE** (0.1 mM) with CB[7] (2.00 mM) in water.

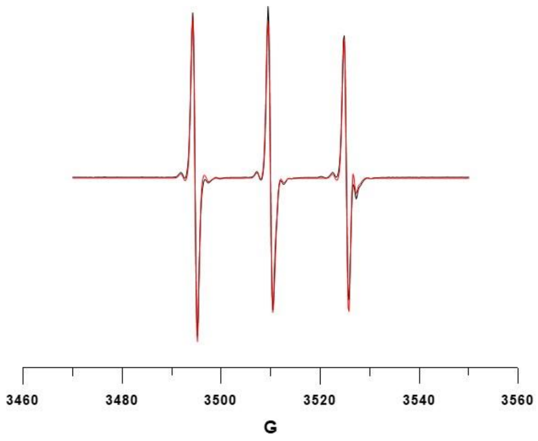

Figure S21. Experimental and simulated EPR spectra of **TEMPONE** (0.1 mM) with CB[7] (2.50 mM) in water.

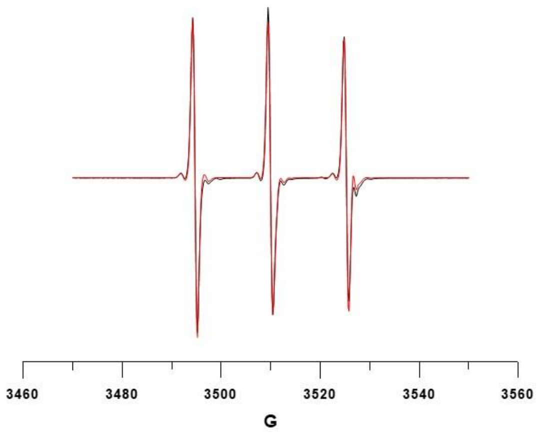

Figure S22. Experimental and simulated EPR spectra of **TEMPONE** (0.1 mM) with CB[7] (3.00 mM) in water.

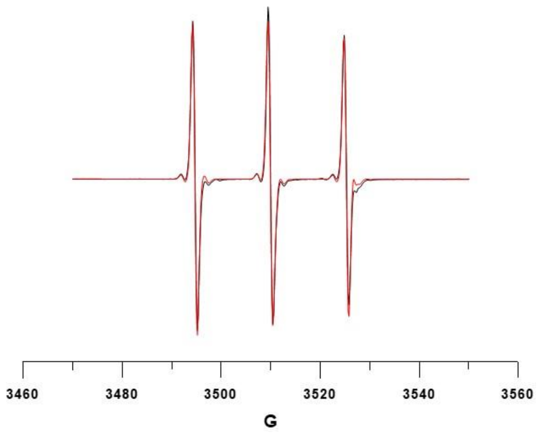

Figure S23. Experimental and simulated EPR spectra of **TEMPONE** (0.1 mM) with CB[7] (5.00 mM) in water.

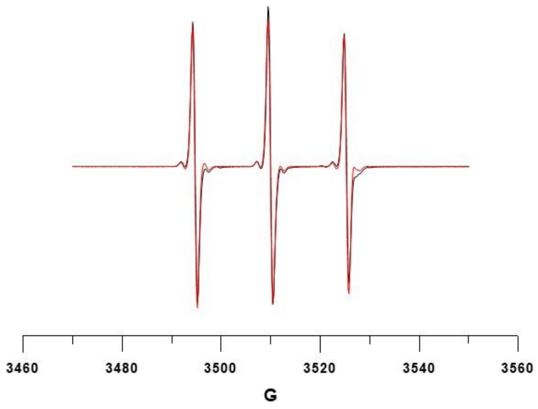

Figure S24. Experimental and simulated EPR spectra of **TEMPONE** (0.1 mM) with CB[7] (7.00 mM) in water.

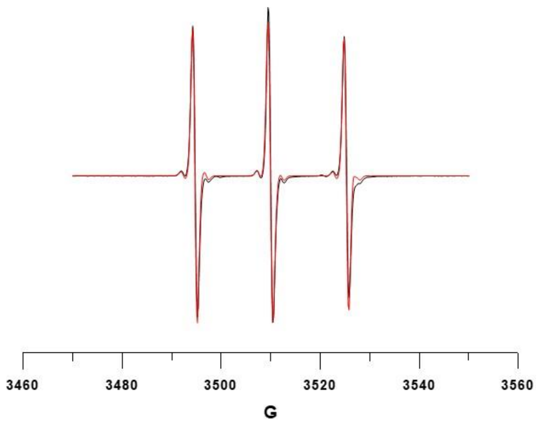

Figure S25. Experimental and simulated EPR spectra of **TEMPONE** (0.1 mM) with CB[7] (9.50 mM) in water.

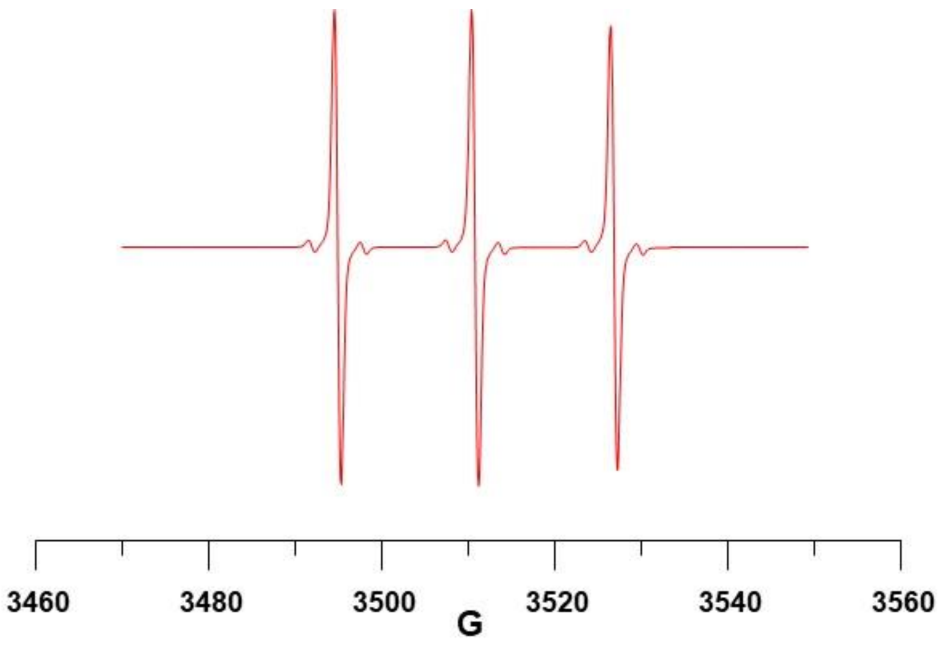

Figure S26. Simulated EPR spectrum of **TEMPONE** in water.

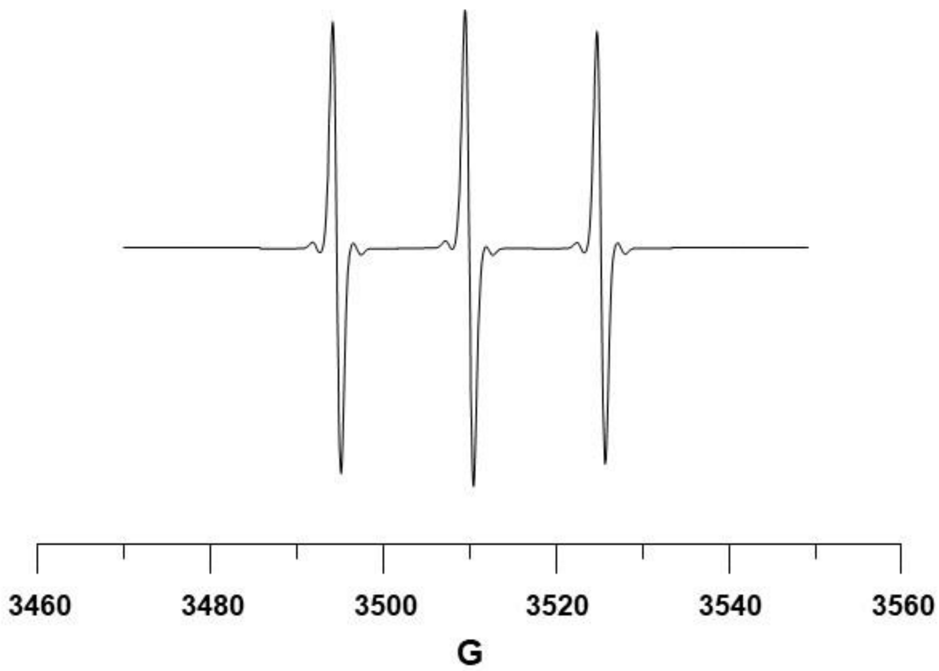

Figure S27. Simulated EPR spectrum of the **TEMPONE•**CB[7]$_1$ complex in water.

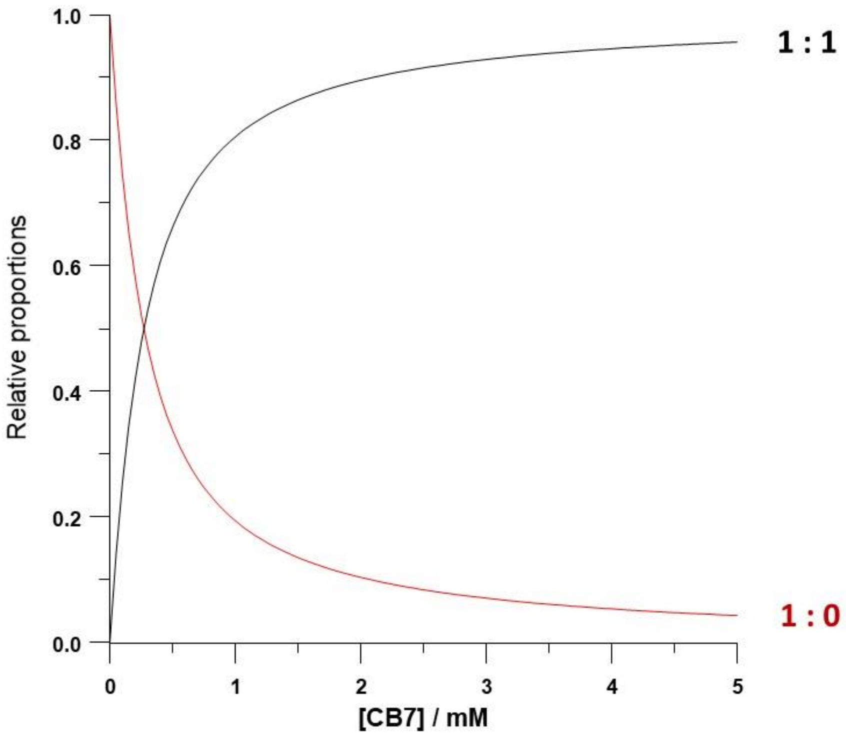

Figure S28. Distribution curve of each species based on the 1:1 guest:host assumption. The red curve is for the nitroxide alone (1:0), the black curve is for the 1:1 complex (1:1).

5/ NMR spectra of reduced TEMPONE with CB[7].

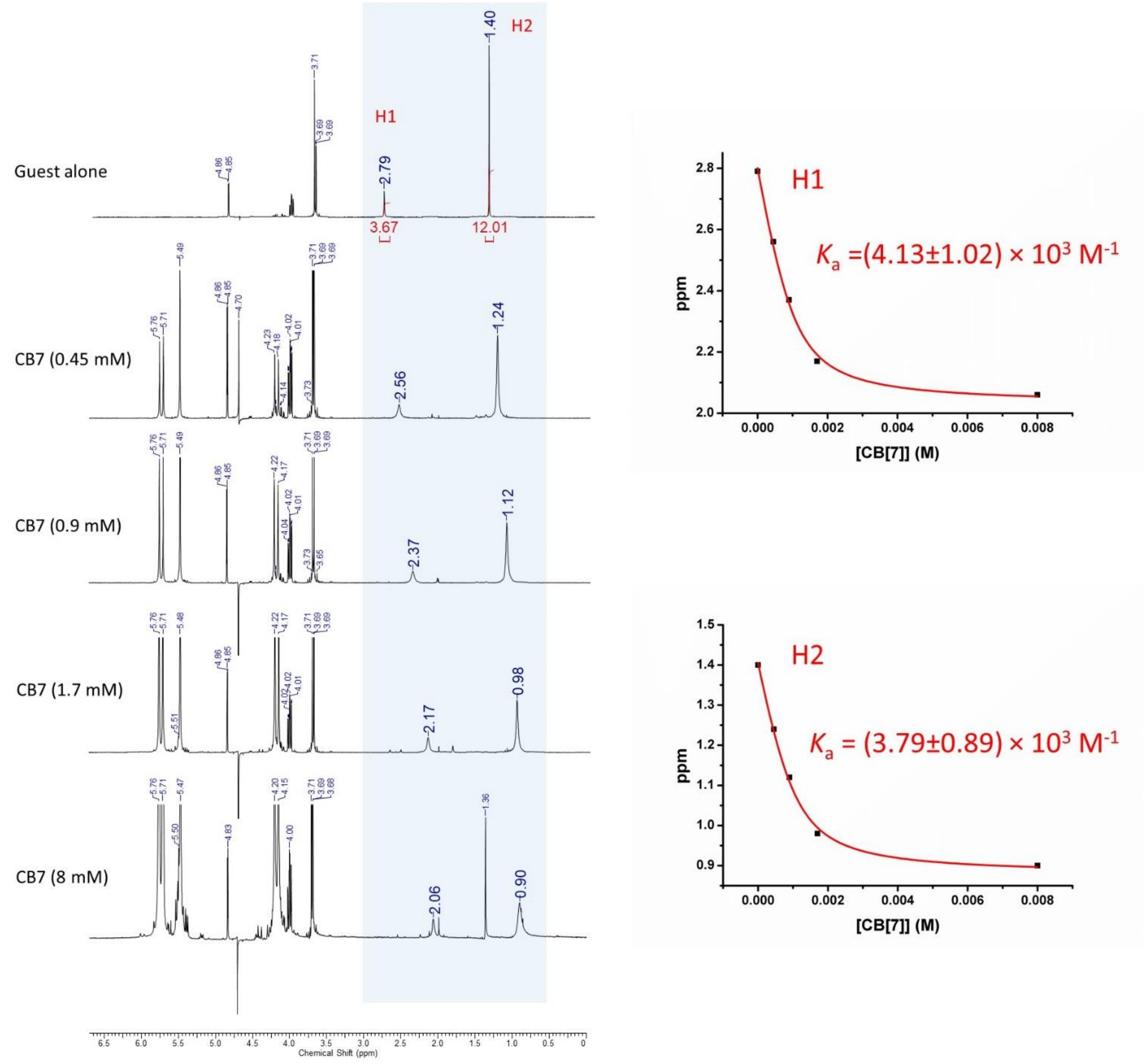

Figure S29. ^{1}H NMR spectra of reduced (ascorbic acid, constant concentration of 8 mM) **TEMPONE** (final constant concentration of 1 mM) in the presence of increasing amounts of CB[7] (concentration indicated on the left). Because the exchange is fast with respect to the NMR timescale, several spectra are necessary and a fit using a typical 1:1 binding mode was performed affording an averaged K_a = 3960 M^{-1}. The *reduced* **TEMPONE** is the corresponding diamagnetic molecule with a hydroxylamine (N-OH) instead of a nitroxide (N-O•) due to the presence of ascorbic acid as a reductant.

6/ EPR titration (black spectra) and simulations (red spectra) for bTbk with CB[8].

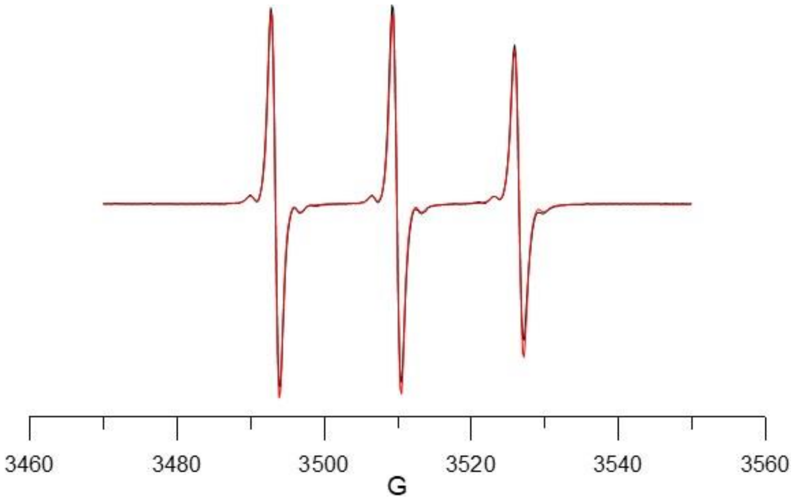

Figure S30. Experimental and simulated EPR spectra of **bTbk** (0.05 mM) in water.

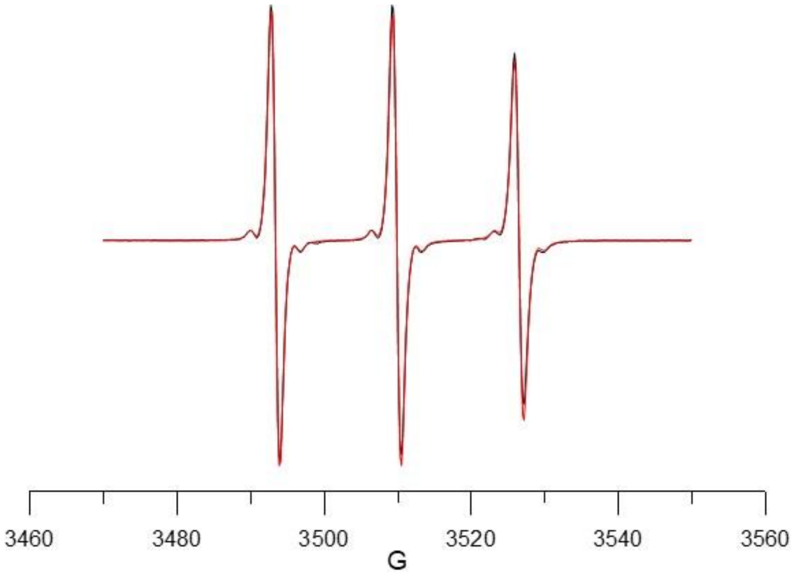

Figure S31. Experimental and simulated EPR spectra of **bTbk** (0.05 mM) with CB[8] (0.005 mM) in water.

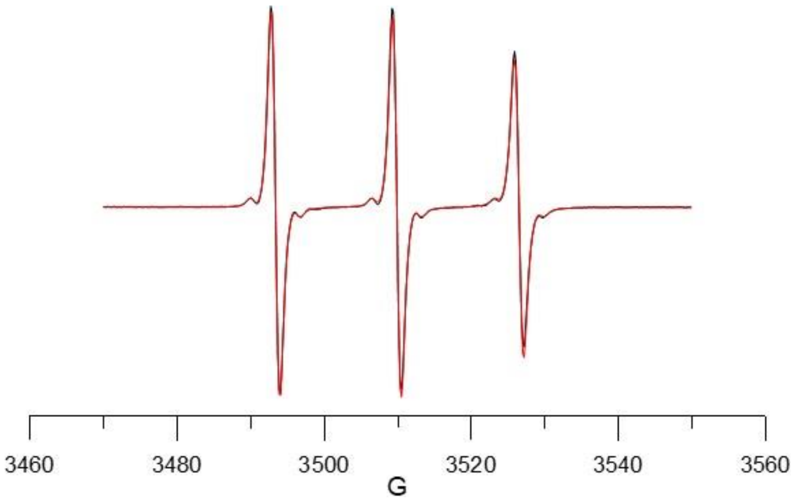

Figure S32. Experimental and simulated EPR spectra of **bTbk** (0.05 mM) with CB[8] (0.010 mM) in water.

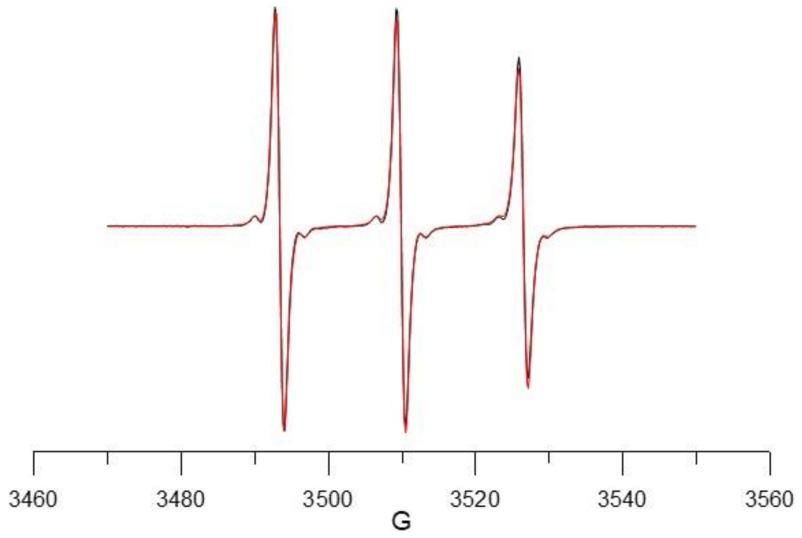

Figure S33. Experimental and simulated EPR spectra of **bTbk** (0.05 mM) with CB[8] (0.015 mM) in water.

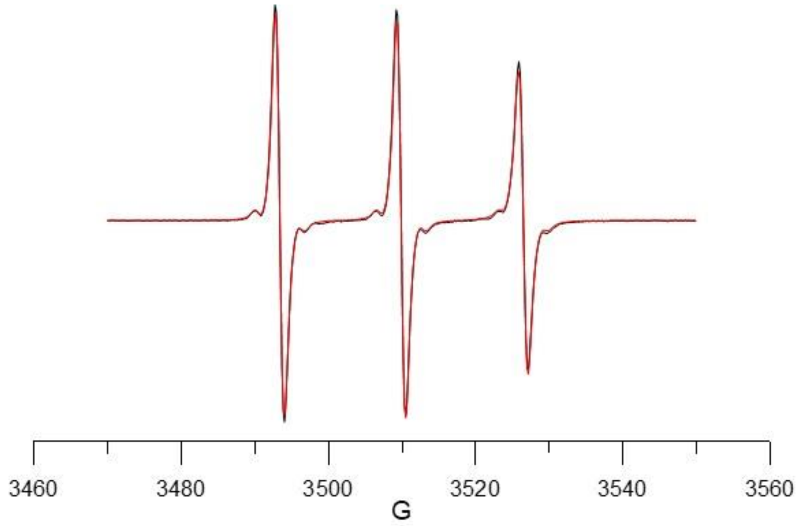

Figure S34. Experimental and simulated EPR spectra of **bTbk** (0.05 mM) with CB[8] (0.020 mM) in water.

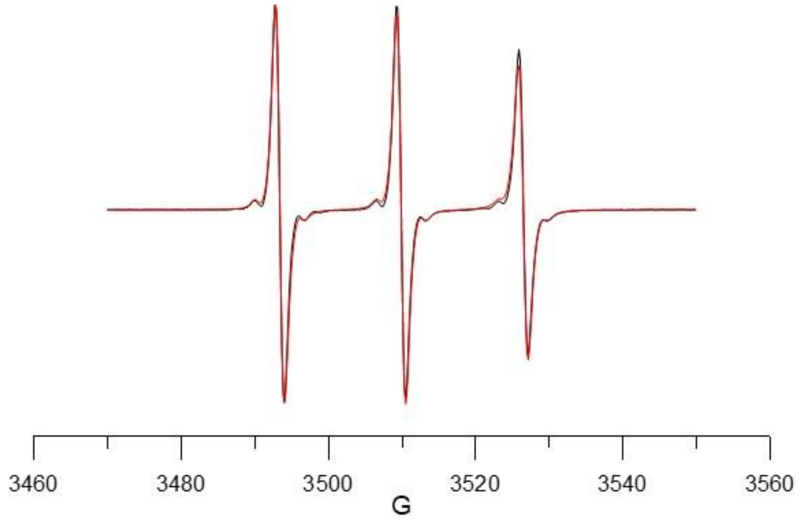

Figure S35. Experimental and simulated EPR spectra of **bTbk** (0.05 mM) with CB[8] (0.025 mM) in water.

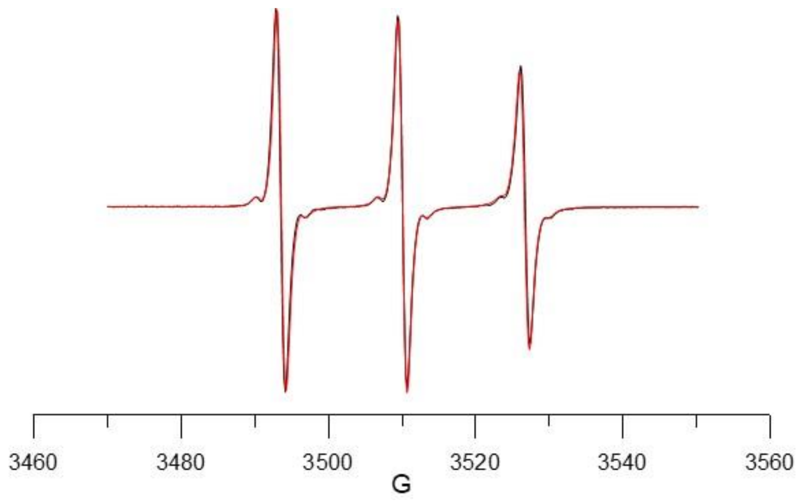

Figure S36. Experimental and simulated EPR spectra of **bTbk** (0.05 mM) with CB[8] (0.030 mM) in water.

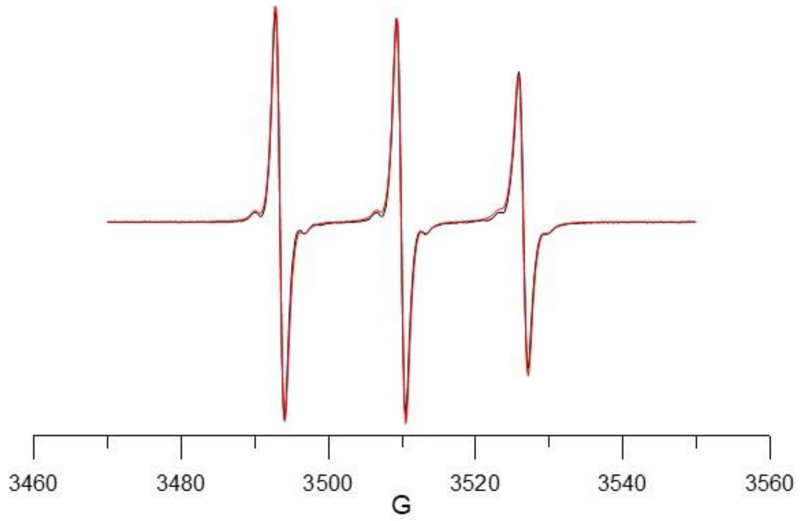

Figure S37. Experimental and simulated EPR spectra of **bTbk** (0.05 mM) with CB[8] (0.035 mM) in water.

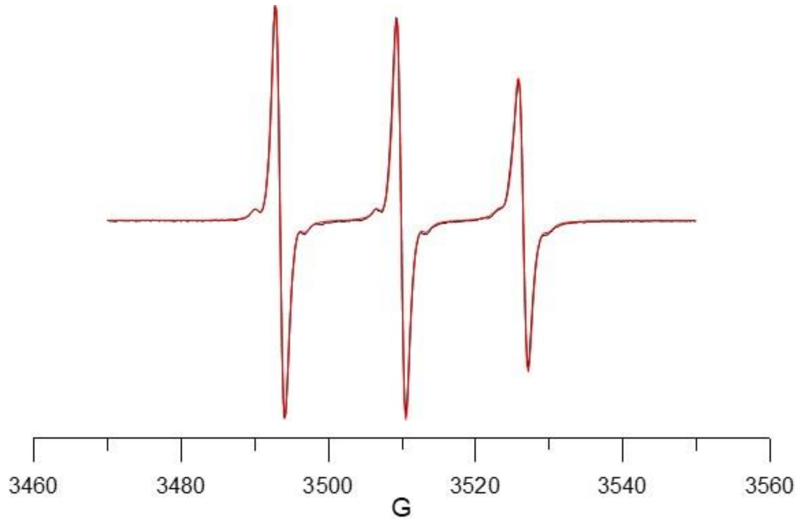

Figure S38. Experimental and simulated EPR spectra of **bTbk** (0.05 mM) with CB[8] (0.040 mM) in water.

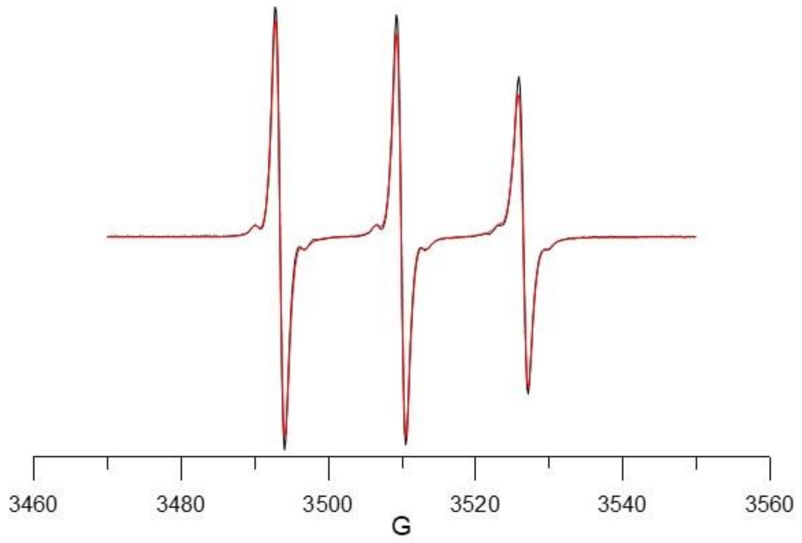

Figure S39. Experimental and simulated EPR spectra of **bTbk** (0.05 mM) with CB[8] (0.045 mM) in water.

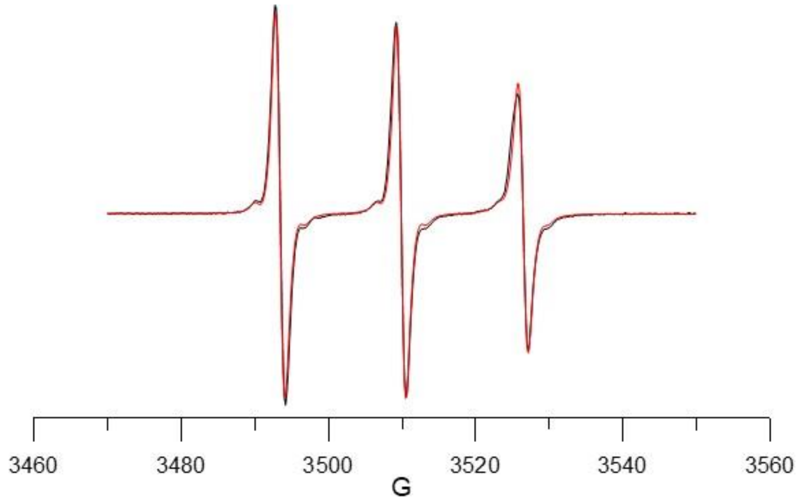

Figure S40. Experimental and simulated EPR spectra of **bTbk** (0.05 mM) with CB[8] (0.050 mM) in water.

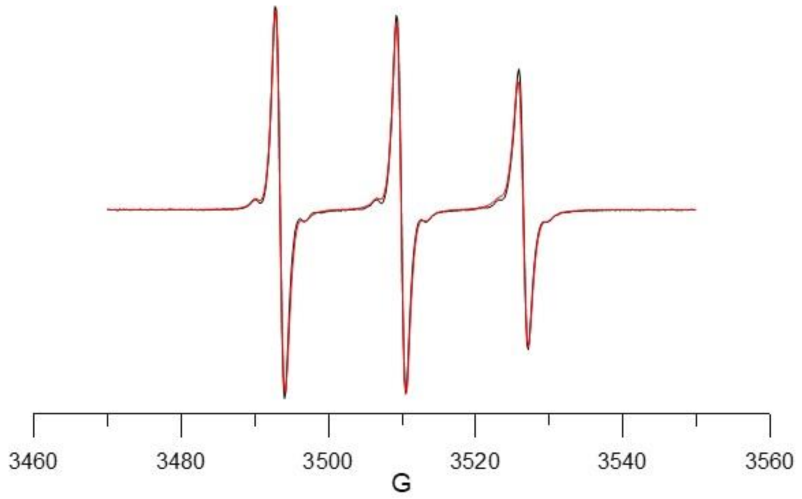

Figure S41. Experimental and simulated EPR spectra of **bTbk** (0.05 mM) with CB[8] (0.055 mM) in water.

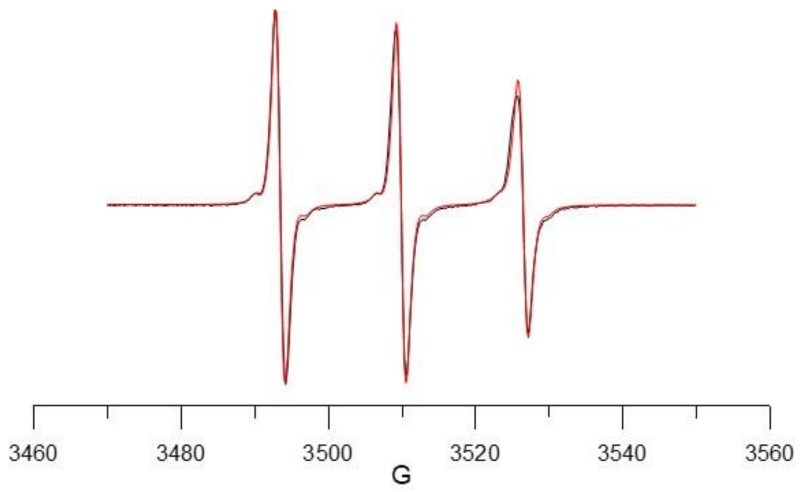

Figure S42. Experimental and simulated EPR spectra of **bTbk** (0.05 mM) with CB[8] (0.060 mM) in water.

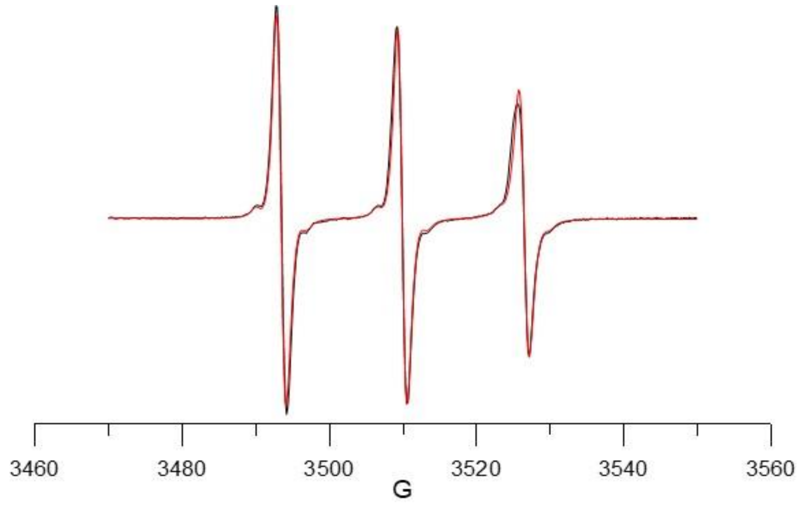

Figure S43. Experimental and simulated EPR spectra of **bTbk** (0.05 mM) with CB[8] (0.065 mM) in water.

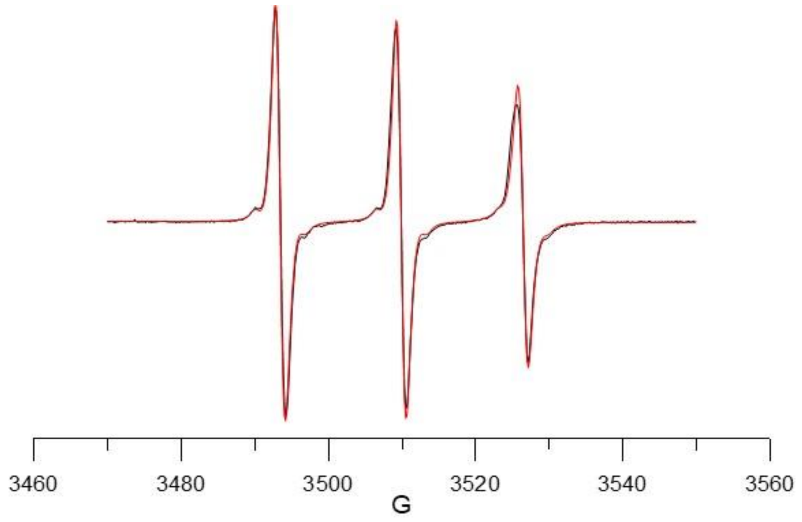

Figure S44. Experimental and simulated EPR spectra of **bTbk** (0.05 mM) with CB[8] (0.070 mM) in water.

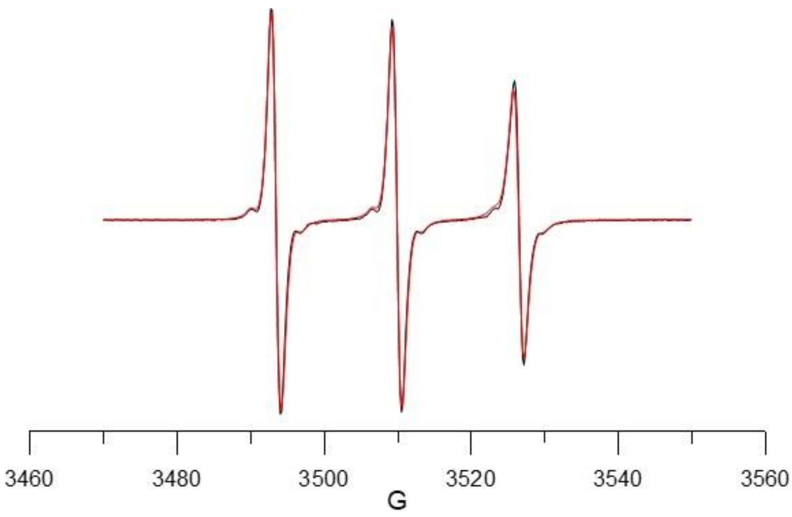

Figure S45. Experimental and simulated EPR spectra of **bTbk** (0.05 mM) with CB[8] (0.075 mM) in water.

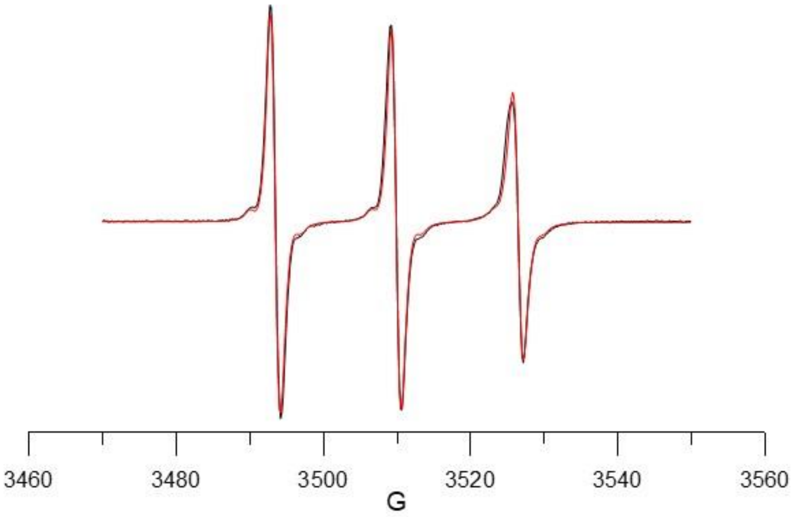

Figure S46. Experimental and simulated EPR spectra of **bTbk** (0.05 mM) with CB[8] (0.080 mM) in water.

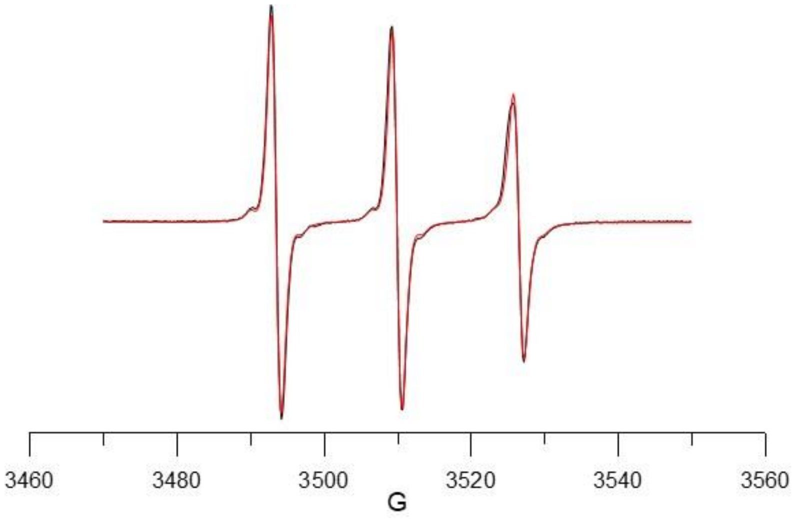

Figure S47. Experimental and simulated EPR spectra of **bTbk** (0.05 mM) with CB[8] (0.085 mM) in water.

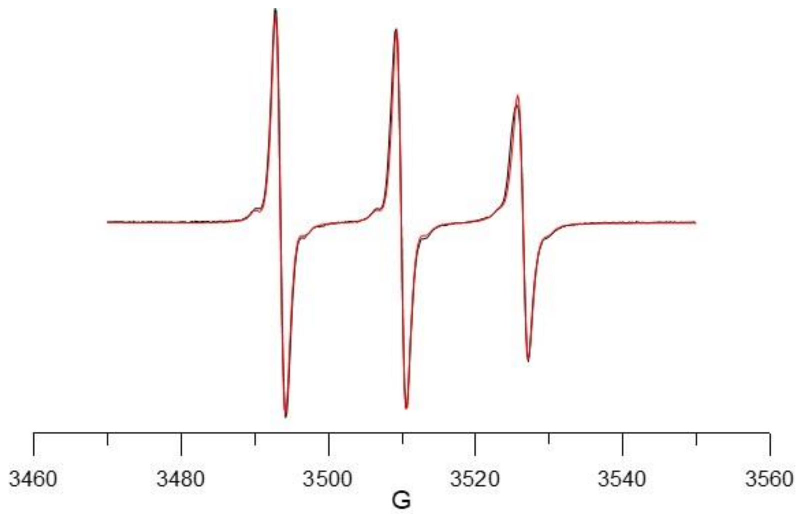

Figure S48. Experimental and simulated EPR spectra of **bTbk** (0.05 mM) with CB[8] (0.090 mM) in water.

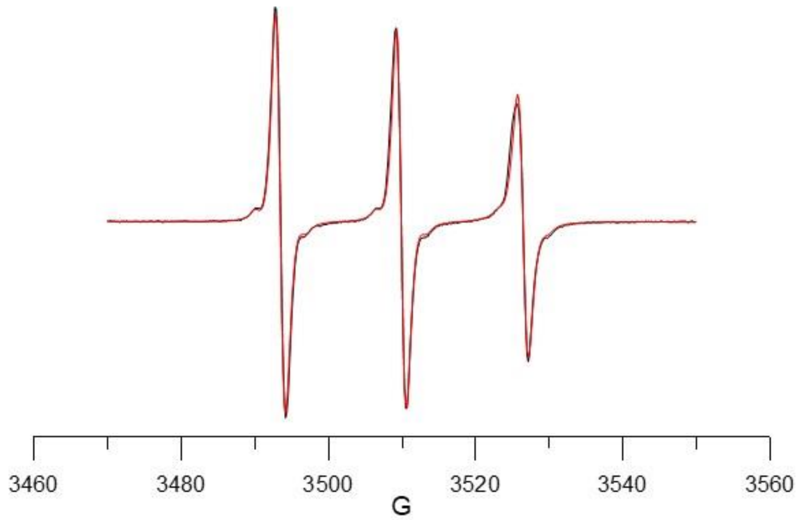

Figure S49. Experimental and simulated EPR spectra of **bTbk** (0.05 mM) with CB[8] (0.095 mM) in water.

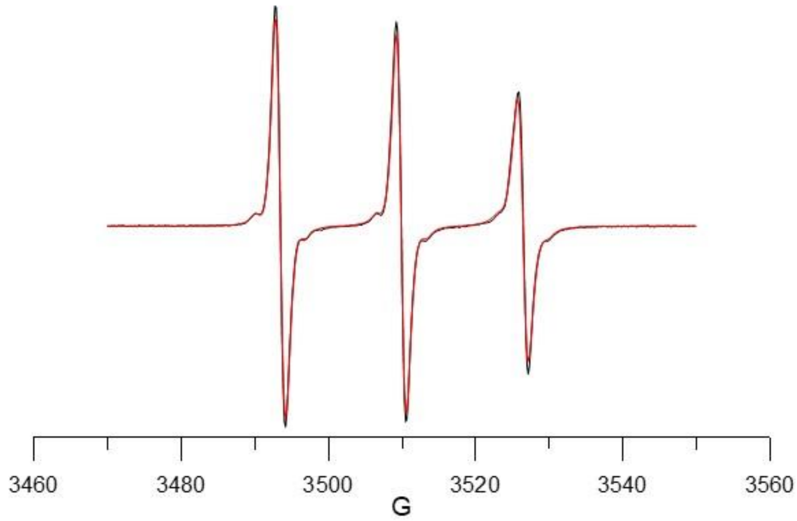

Figure S50. Experimental and simulated EPR spectra of **bTbk** (0.05 mM) with CB[8] (0.100 mM) in water.

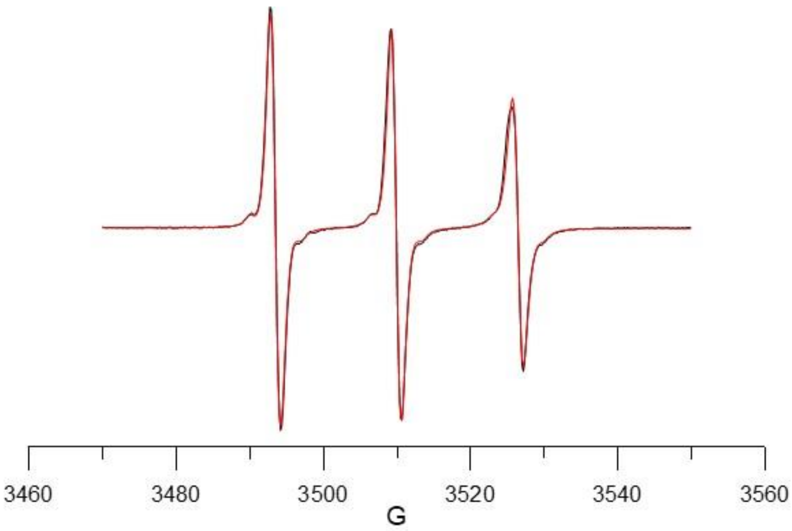

Figure S51. Experimental and simulated EPR spectra of **bTbk** (0.05 mM) with CB[8] (0.110 mM) in water.

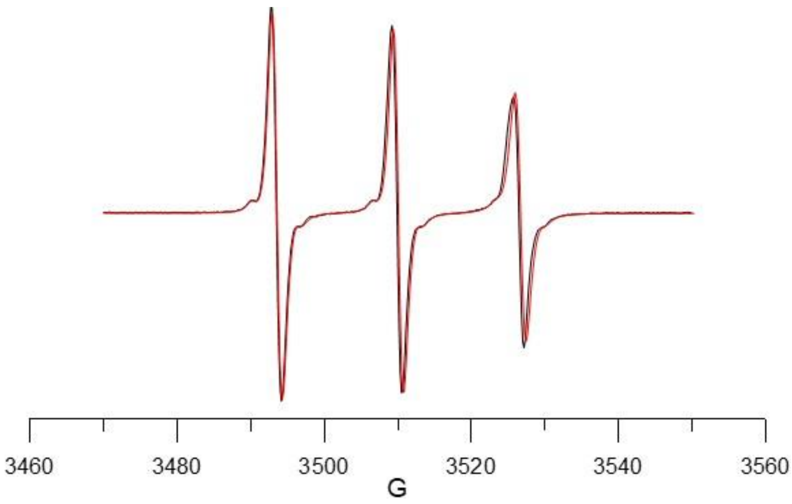

Figure S52. Experimental and simulated EPR spectra of **bTbk** (0.05 mM) with CB[8] (0.120 mM) in water.

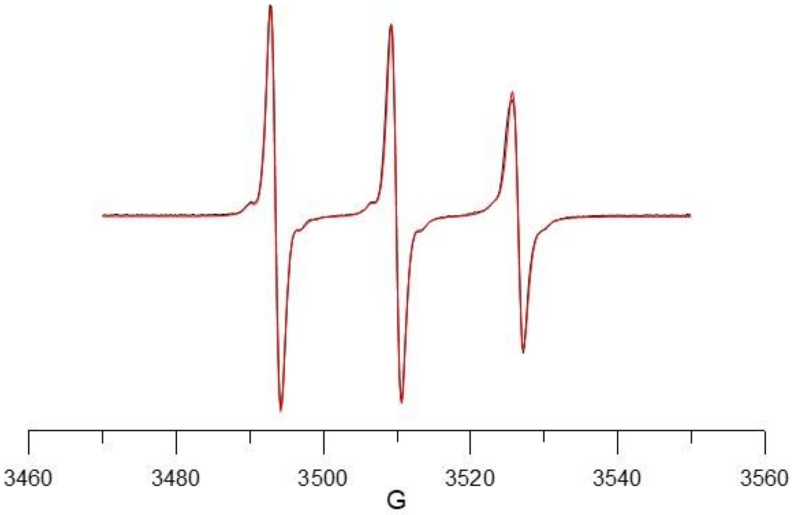

Figure S53. Experimental and simulated EPR spectra of **bTbk** (0.05 mM) with CB[8] (0.130 mM) in water.

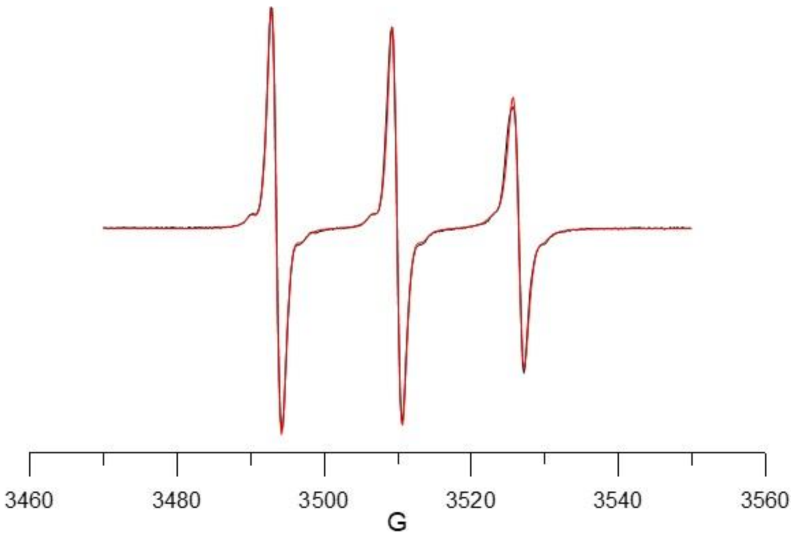

Figure S54. Experimental and simulated EPR spectra of **bTbk** (0.05 mM) with CB[8] (0.140 mM) in water.

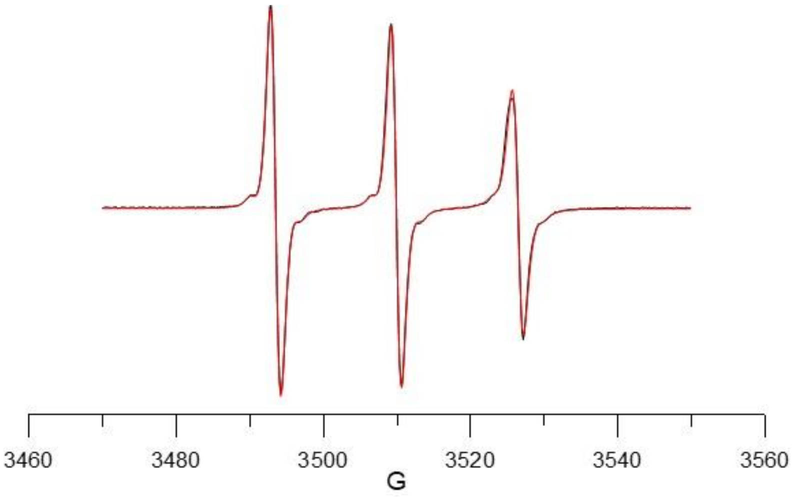

Figure S55. Experimental and simulated EPR spectra of **bTbk** (0.05 mM) with CB[8] (0.150 mM) in water.

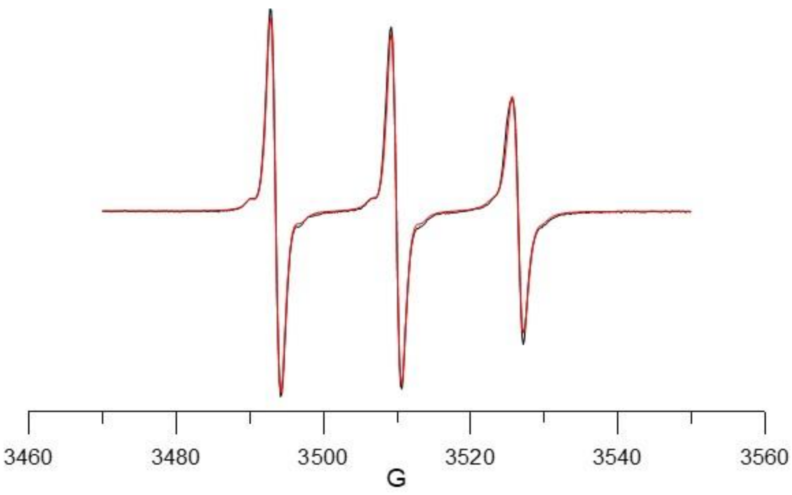

Figure S56. Experimental and simulated EPR spectra of **bTbk** (0.05 mM) with CB[8] (0.160 mM) in water.

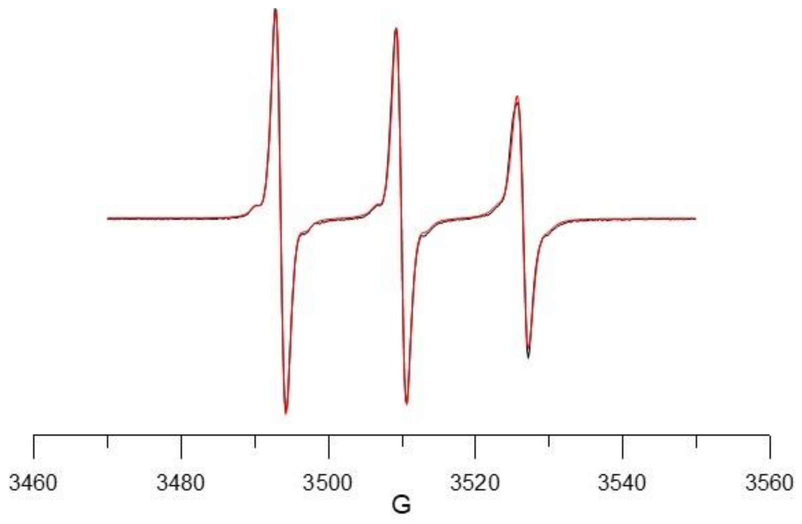

Figure S57. Experimental and simulated EPR spectra of **bTbk** (0.05 mM) with CB[8] (0.170 mM) in water.

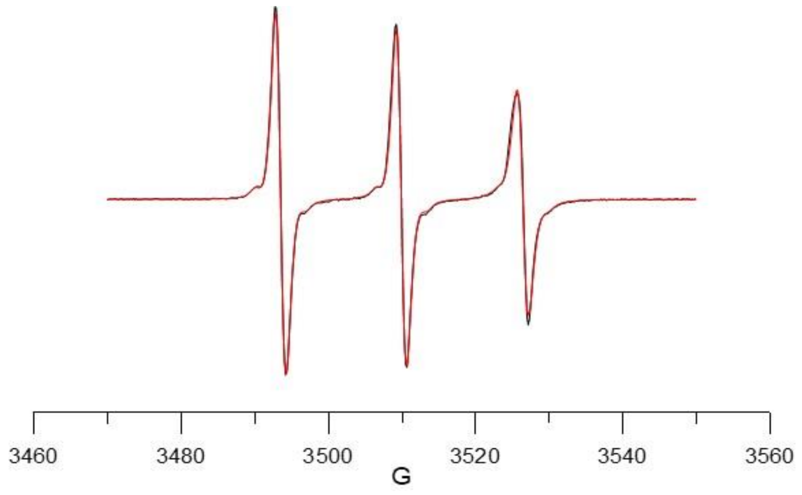

Figure S58. **E**xperimental and simulated EPR spectra of **bTbk** (0.05 mM) with CB[8] (0.180 mM) in water.

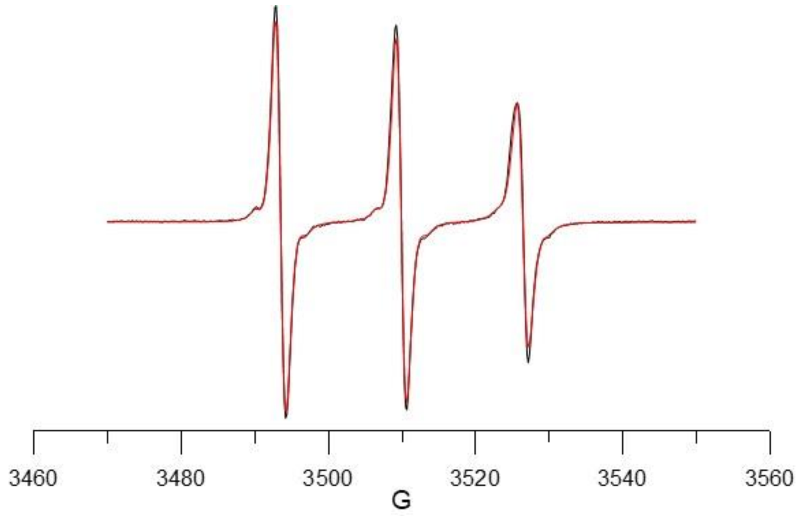

Figure S59. Experimental and simulated EPR spectra of **bTbk** (0.05 mM) with CB[8] (0.190 mM) in water.

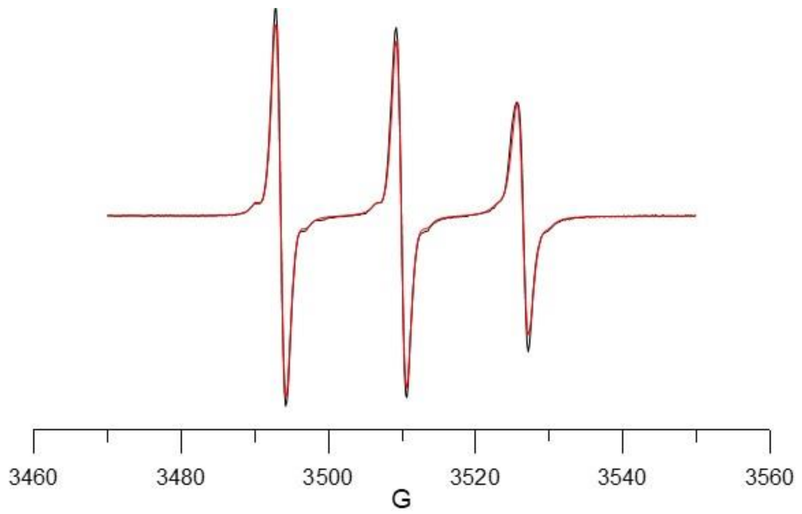

Figure S60. Experimental and simulated EPR spectra of **bTbk** (0.05 mM) with CB[8] (0.20 mM) in water.

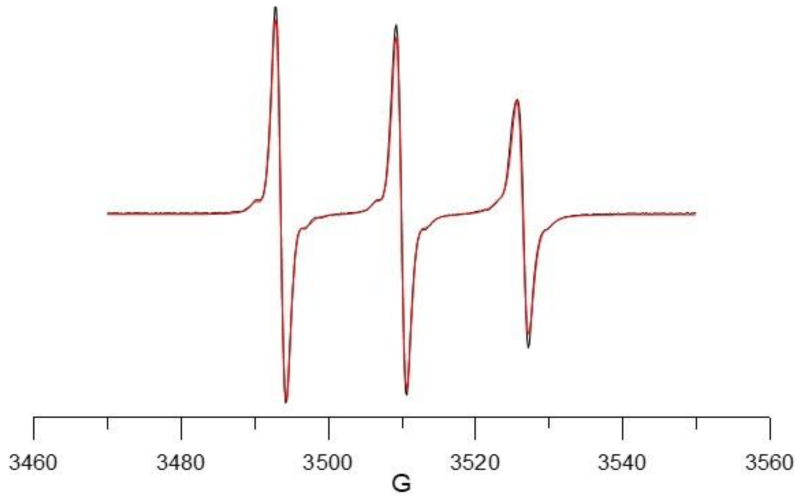

Figure S61. Experimental and simulated EPR spectra of **bTbk** (0.05 mM) with CB[8] (0.25 mM) in water.

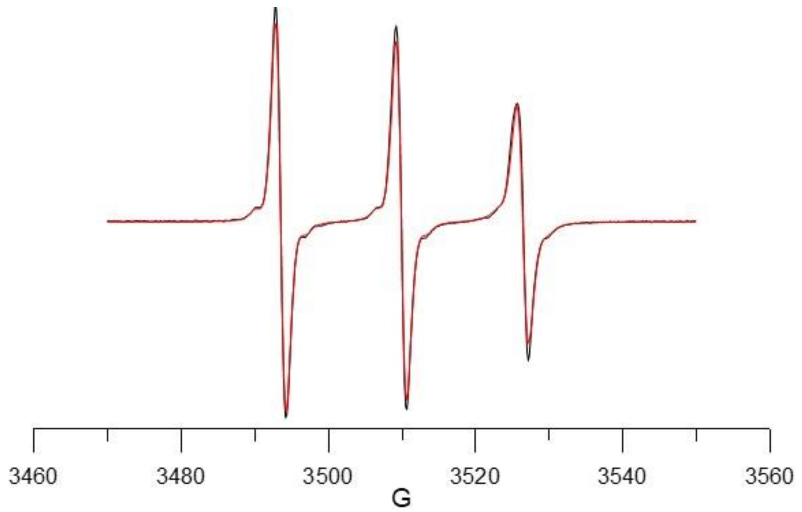

Figure S62. Experimental and simulated EPR spectra of **bTbk** (0.05 mM) with CB[8] (0.40 mM) in water.

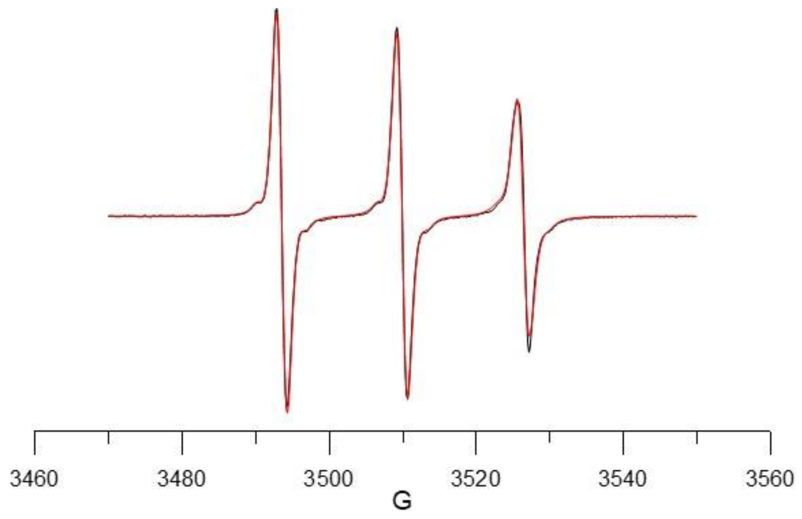

Figure S63. Experimental and simulated EPR spectra of **bTbk** (0.05 mM) with CB[8] (0.50 mM) in water.

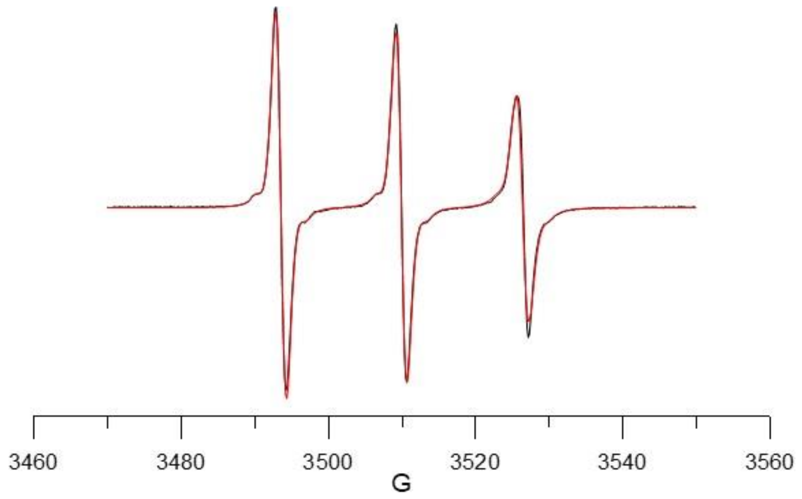

Figure S64. Experimental and simulated EPR spectra of **bTbk** (0.05 mM) with CB[8] (0.80 mM) in water.

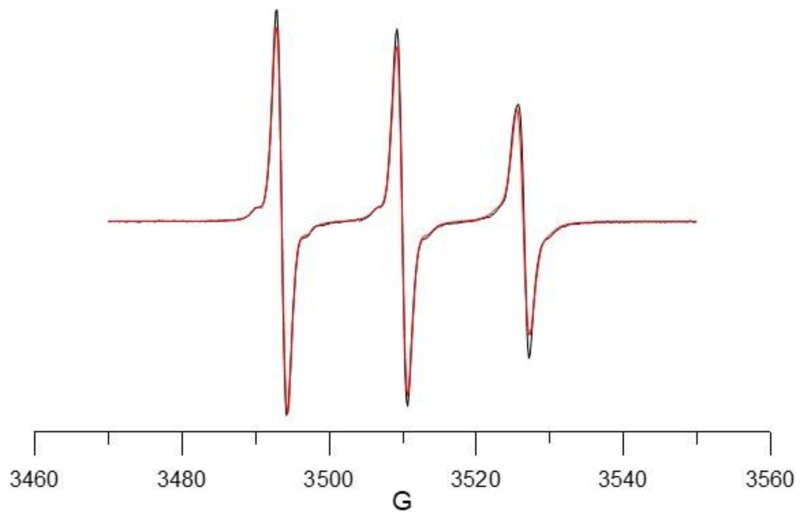

Figure S65. Experimental and simulated EPR spectra of **bTbk** (0.05 mM) with CB[8] (1.0 mM) in water.

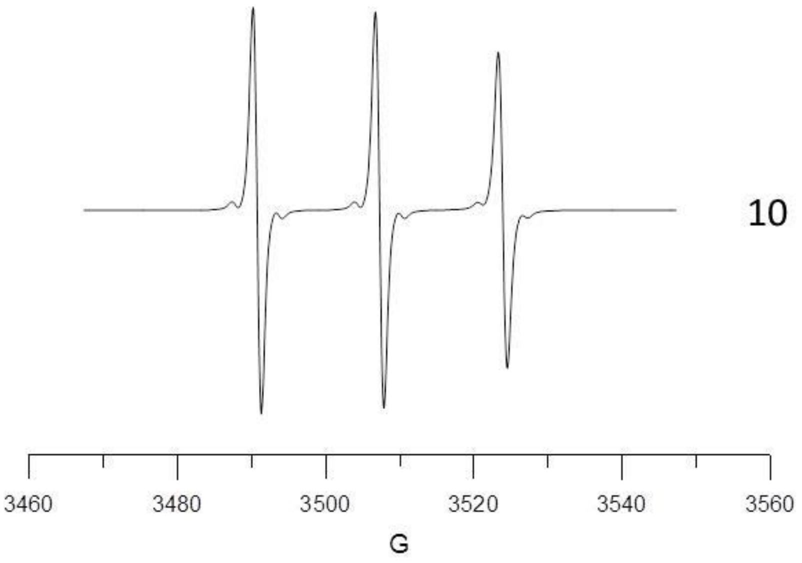

Figure S66. Simulated EPR spectrum of **bTbk** in water.

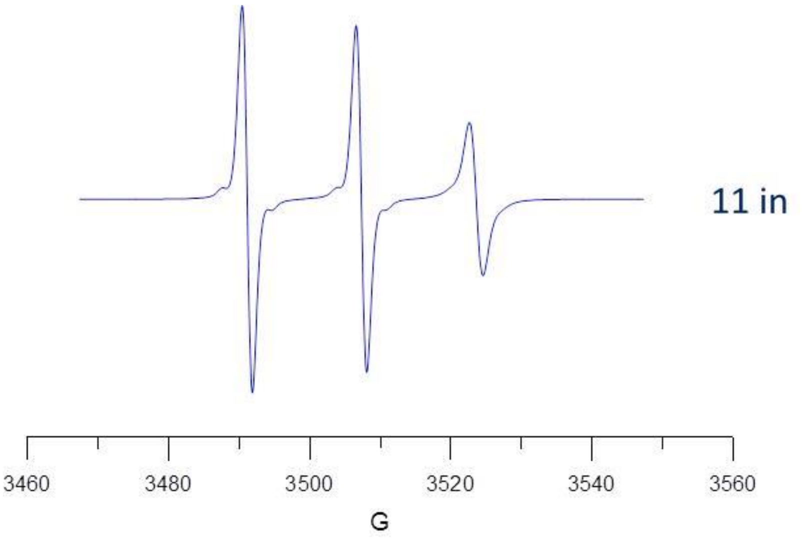

Figure S67. Simulated EPR spectrum of **bTbk** in water (complexed TEMPO part).

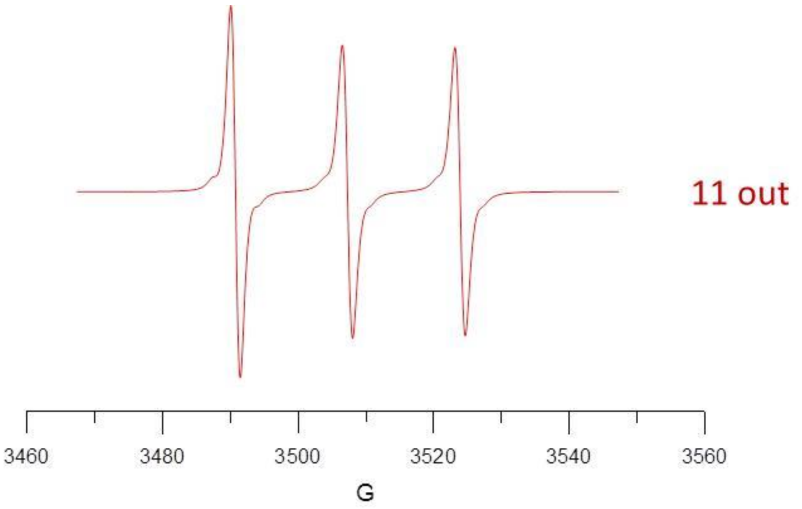

Figure S68. Simulated EPR spectrum of **bTbk** in water (free TEMPO part).

Results of the simulations of EPR spectra for the bTbk•CB[8] titration.

Shuttling model, 10 stands for the free radical, 11-in for the TEMPO group included in CB[8] and 11-out for the other TEMPO, bulk exposed. Since one TEMPO is outside of the cavity its EPR parameters were fixed as the values obtained for 10, only the three relaxation parameters were allowed to change. Overall regression parameter = 0.9959.

	10	11-IN	11-OUT
a_N	16.59	16.25	16.59*
a_{C13}	5.8	6.0	5.8*
α	0.406	0.622	0.897
β	0.057	0.308	0.075
γ	0.047	0.245	-0.064

*Fixed values.

7/ NMR spectra of reduced bTbk with CB[8].

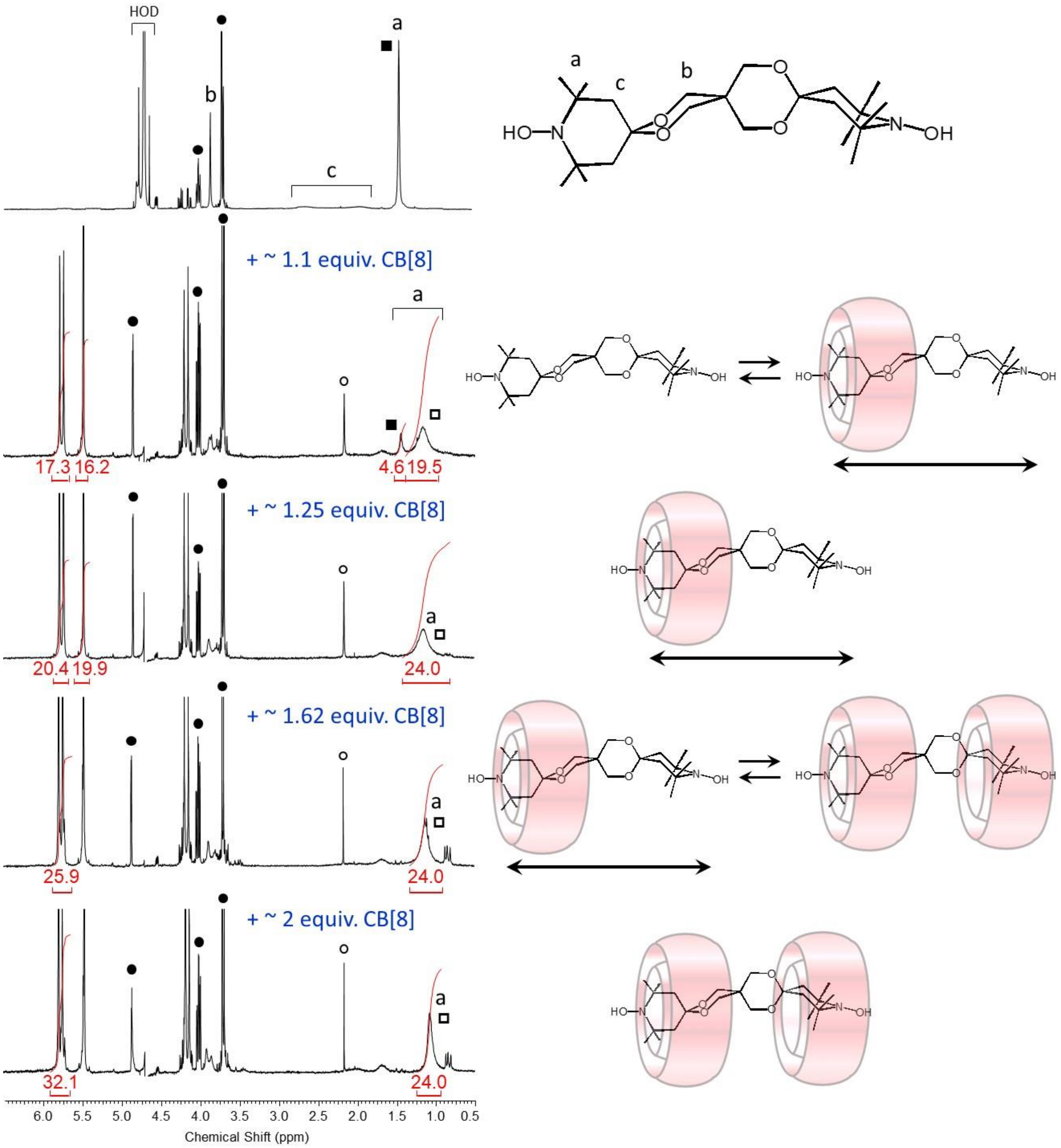

Figure S69. ^{1}H NMR spectra of reduced ([ascorbic acid] = 4 mM: ●) **bTbk** (0.5 mM) with increasing amounts of CB[8] in D_2O (○ corresponds to the signal of acetone, ■ is for free guest and □ is for included guest). Proposed complexes and corresponding equilibria are on the right part.

Since the signal corresponding to the 8 methyl groups is shifted upfield at 1.25 equiv. of CB[8] added, we propose a shuttling motion of the host to explain why all the methyl groups are affected. At 2 equiv. of host, the spectrum is very similar but integrals indicate that 2 CB[8] are dissolved suggesting a 1:2 (guest:host) complex stoichiometry.

8/ Competition experiments (EPR) for the binding of bTbk with CB[8].

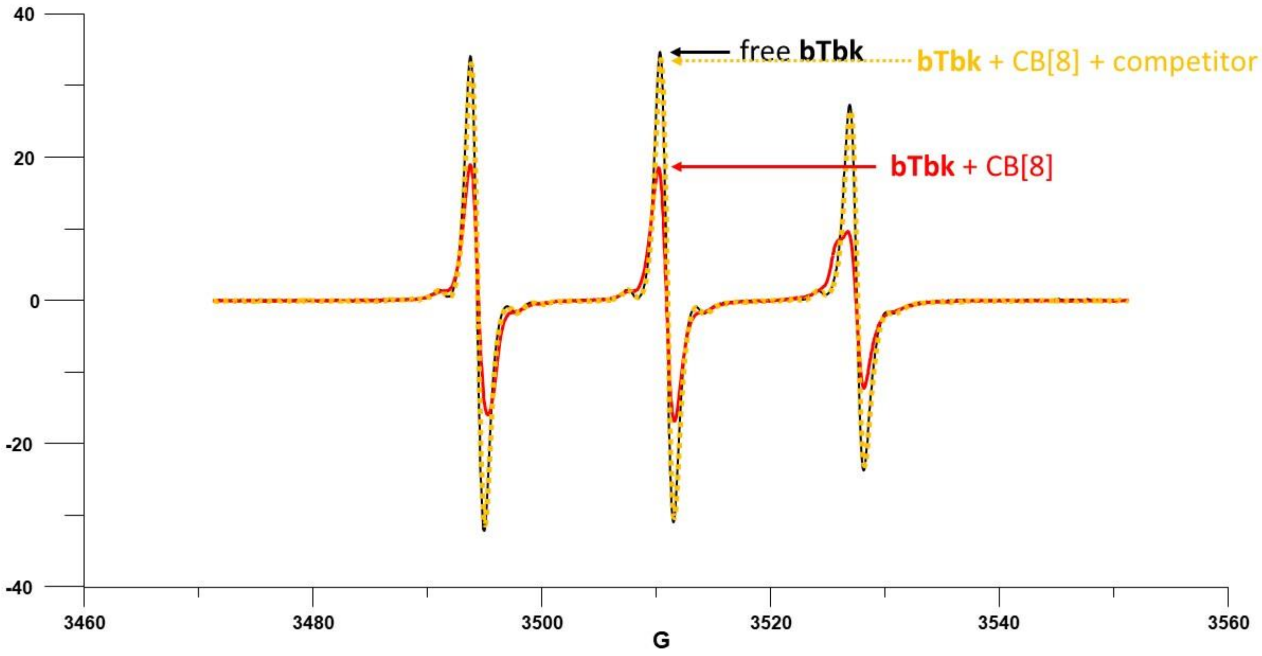

Figure S70. EPR spectra of **bTbk** (black spectrum), **bTbk** with CB[8] (red spectrum) and **bTbk** with CB[8] in the presence of a competitor (amantadine hydrochloride, orange dashed line spectrum). Concentrations are as follows: [**bTbk**] = 50 μM, [CB8] = 400 μM and [competitor] = 4 mM, all in distilled water.

9/ References.

[1] Matsuki, Y.; Maly, T.; Ouari, O.; Karoui, H.; Le Moigne, F.; Rizzato, E.; Lyubenova, S.; Herzfeld, J.; Prisner, T.; Tordo, P.; Griffin, R. G. Dynamic Nuclear Polarization with a Rigid Biradical, *Angew. Chem. Int. Ed.* **2009**, *48*, 4996–5000. DOI: 10.1002/anie.200805940.

[2] Day, A.; Arnold, A. P.; Blanch, R. J.; Snushall, B. Controlling Factors in the Synthesis of Cucurbituril and Its Homologues, *J. Org. Chem.* **2001**, *66*, 8094–8100. DOI: 10.1021/jo015897c.

[3] Kim, J.; Jung, I.-S.; Kim, S.-Y.; Lee, E.; Kang, J.-K.; Sakamoto, S.; Yamaguchi, K.; Kim, K. New Cucurbituril Homologues: Syntheses, Isolation, Characterization, and X-ray Crystal Structures of Cucurbit[*n*]uril (*n* = 5, 7, and 8), *J. Am. Chem. Soc.* **2000**, *122*, 540–541. DOI: 10.1021/ja993376p.

[4] Bardelang, D.; Udachin, K. A.; Leek, D. M.; Margeson, J. C.; Chan, G.; Ratcliffe, C. I.; Ripmeester, J. A. Cucurbit[*n*]urils (*n* = 5–8): A Comprehensive Solid State Study, *Cryst. Growth Des.* **2011**, *11*, 5598–5614. DOI: 10.1021/cg201173j.
